# Correcting for publication bias in a meta-analysis with the *p*-uniform* method

**DOI:** 10.3758/s13423-025-02812-4

**Published:** 2026-02-27

**Authors:** Robbie C. M. van Aert, Marcel A. L. M. van Assen

**Affiliations:** 1 Department of Methodology and Statistics, Tilburg University, PO Box 90153, 5000 LE Tilburg, the Netherlands; 2 Department of Sociology, Utrecht University, Utrecht, the Netherlands

**Keywords:** Publication bias, Meta-analysis, *P*-uniform, Selection model approach

## Abstract

Publication bias is a major threat to the validity of a meta-analysis, resulting in overestimated effect sizes. We propose a generalization and improvement of the publication bias method *p-*uniform called *p*-uniform*. *P*-uniform* improves upon *p*-uniform in three ways, as it (i) entails a more efficient estimator, (ii) eliminates the overestimation of effect size caused by between-study variance in true effect sizes, and (iii) enables estimating and testing for the presence of the between-study variance. We compared the statistical properties of *p*-uniform* with *p-*uniform, two implementations of the three-parameter selection model (3PSM) approach, and the random-effects model. Statistical properties of *p*-uniform* and 3PSM were comparable and generally outperformed *p-*uniform and the random-effects model if publication bias was present. We explain that *p-*uniform* uses a more parsimonious model than 3PSM and demonstrate that both methods estimate average effect size and between-study variance rather well with ten or more studies in the meta-analysis when publication bias is not extreme. We re-analyze the data of two published meta-analyses using *p*-uniform, *p-*uniform*, and 3PSM to illustrate the impact of publication bias on the results. We also offer recommendations for applied researchers, and we share R code in an R package as well as an easy-to-use web application for applying *p*-uniform*.

## Introduction

Effect sizes from multiple primary studies can be statistically combined by means of a meta-analysis in order to obtain a quantitative summary of the studied relationship. A major threat to the validity of a meta-analysis is publication bias (Borenstein, Hedges, Higgins, & Rothstein, 2009; Rothstein, Sutton, & Borenstein, 2005). Publication bias refers to situations where the published literature is not a representative reflection of the population of completed studies (Rothstein et al., 2005). For example, this may imply that studies with statistically significant results are more likely to be published than studies with statistically nonsignificant results. Publication bias is not only caused by reviewers and editors who are reluctant to accept studies without statistically significant results, but also by researchers who do not submit studies with nonsignificant results (Cooper, DeNeve, & Charlton, 1997; Coursol & Wagner, 1986). The consequences of publication bias are severe and may hamper scientific progress, because publication bias results in overestimated effect sizes in primary studies when statistically significant effect sizes are favored over nonsignificant effect sizes. Combining these overestimated effect sizes in a meta-analysis yields an overestimated meta-analytic effect size estimate as well (Kraemer, Gardner, Brooks, & Yesavage, 1998; Lane & Dunlap, 1978).

Evidence for publication bias has been observed in multiple research fields. Fanelli (2010, 2012) studied how often the authors declared having found support for the tested hypothesis in a random sample of published papers from a variety of research fields. In psychiatry and psychology, 90% of the papers concluded that the hypothesis was supported, which was the largest percentage across all included research fields. However, this large percentage is not in line with the estimated average statistical power of approximately 50% (or lower) in psychological research (Bakker, van Dijk, & Wicherts, 2012; Cohen, 1990). Other more direct evidence of publication bias in psychology was found in Franco, Malhotra, and Simonovits (2014). They studied whether experiments in the social sciences were more likely to be published if the authors of these experiments deemed the results to be strong, mixed, or null. Franco et al. (2014) observed evidence that experiments with null or mixed results were less often published compared to strong results.

Mixed evidence for the presence of publication bias has been observed when re-analyzing published meta-analyses. Bartoš et al. (2024) re-analyzed 68,517 meta-analyses from medicine, environmental science, psychology, and economics. They concluded that especially meta-analyses in economics, environmental science, and psychology are affected by publication bias. Other re-analyses of meta-analyses have resulted in less strong evidence for publication bias. For instance, a re-analysis of meta-analyses about post-traumatic stress disorder (Niemeyer et al., 2020) and a re-analysis of meta-analyses in psychology and medicine (van Aert, Wicherts, & van Assen, 2019) concluded that there was weak evidence of mild publication bias. Not observing strong evidence for publication bias in these meta-analyses may be explained by effects being included in these meta-analyses that were not the primary outcome of a study. Publication bias is expected to work on the primary outcome of a study and less on secondary outcomes. Furthermore, evidence for publication bias in meta-analyses in medicine might be weaker due to the longer history of registering clinical trials in this field compared to others (e.g., Dickersin & Rennie, 2003; Simes, 1986).

In this paper, we present a generalization and improvement of *p*-uniform (van Aert, Wicherts, & van Assen, 2016; van Assen, van Aert, & Wicherts, 2015) and *p*-curve (Simonsohn, Nelson, & Simmons, 2014) to correct effect size for publication bias when estimating effect size. *p*-uniform and *p*-curve are based on the same methodology but slightly differ in implementation (for a comparison of the two methods, see van Aert et al., 2016). These methods use the statistical principle that the *p*-values should be uniformly distributed at the true effect size.

Three major drawbacks of *p*-uniform and *p*-curve in their current implementation are that (i) the methods only use statistically significant effect sizes, which makes the methods inefficient (i.e., estimates often have large variance); (ii) effect size estimates are positively biased in the presence of between-study variance in true effect sizes (Carter et al., 2019; McShane et al., 2016; van Aert et al., 2016);^1^ and (iii) they do not estimate and test for the presence of this between-study variance. Our generalized method called *p*-uniform* solves all three drawbacks. Statistically nonsignificant effect sizes are also included in the estimation with *p*-uniform*, (i) making it a more efficient estimator than *p*-uniform, (ii) eliminating the overestimation of effect size in case of between-study variance in true effect sizes, and (iii) enabling estimation and testing for the presence of the between-study variance in true effect sizes.

*P*-uniform, *p*-curve, and the newly proposed *p-*uniform* can be seen as selection model approaches, which are a wide class of approaches to correct for publication bias in a meta-analysis. Selection model approaches combine two models to correct for publication bias: an effect size model and a selection model. The *effect size model* is the distribution of primary studies’ effect sizes in the absence of publication bias and the *selection model* determines how the effect size model is affected by publication bias (Hedges & Vevea, 2005). Several types of selection model approaches have been proposed, varying from approaches that estimate the selection model to those that assume a specific selection model and from frequentist to Bayesian approaches (Andrews & Kasy, 2019; Cleary & Casella, 1997; Copas & Shi, 2000; Iyengar & Greenhouse, 1988a; Kicinski, 2013; Vevea & Woods, 2005). The Online Supplementary Materials (OSM; Supplement 1, https://osf.io/jngwk?view_only=c17ce4ec24b748e7b2dff33dcd42942e) provide a general overview of selection models.

The goal of this paper is twofold. First, we generalize *p-*uniform to *p*-uniform*. Second, we examine the statistical properties of *p*-uniform*, *p*-uniform, and the three-parameter selection model (3PSM) in a simulation study and illustrate the differences between the methods when applying these to two published meta-analyses. We compare *p*-uniform* with *p*-uniform and 3PSM, because (i) the comparison allows us to examine the conditions where *p*-uniform* is an improvement over *p*-uniform, (ii) it is assumed in 3PSM that the selection model is unknown and has to be estimated, which is more realistic than other methods (Vevea & Woods, 2005) that assume that the selection model is known, (iii) 3PSM was among the best performing methods in recent simulation studies (Carter et al., 2019; McShane et al., 2016), and (iv) 3PSM is frequently applied in practice.

### Selection model approaches and the three-parameter selection model

All selection method approaches share the common characteristic that they combine an effect size and selection model to correct for publication bias. The effect size model is usually either the equal-effect (also known as the fixed-effect or common-effect model) or random-effects model. The random-effects model assumes that *k* independent effect size estimates, ${y}_{i}$ with *I* = 1, …, *k*, are extracted from primary studies. The random-effects model can be written as

$${y}_{i}=\mu +{\zeta }_{i}+{\epsilon }_{i}$$

where *μ* is the average true effect size, ${\zeta }_{i}$ is a random effect that denotes the difference between *μ* and the *i*th primary study’s true effect size, and ${\varepsilon }_{i}$ is the *i*th primary study’s sampling error. In the random-effects model, it is commonly assumed that ${\zeta }_{i} \sim N\left(0,{\tau }^{2}\right),$ where *τ*^2^ is the between-study variance in true effects, and ${\varepsilon }_{i}\sim N(0,{\sigma }_{i}^{2})$, where ${\sigma }_{i}^{2}$ is the sampling variance of the *i*th primary study. The ${\zeta }_{i}$ and ${\varepsilon }_{i}$ are assumed to be mutually independent of each other, and ${\sigma }_{i}^{2}$ is estimated in practice and then assumed to be known. If ${\tau }^{2}=0$, there is no between-study variance in the true effect sizes, and the random-effects model simplifies to the equal-effect model.

The selection model is a non-negative weight function that determines the likelihood of a primary study getting included in a meta-analysis (Hedges & Vevea, 2005). The weight function, $w({y}_{i},{\sigma }_{i})$, is combined with the effect size model to get a weighted density of ${y}_{i}$,

1 $$\frac{w({y}_{i},{\sigma }_{i})f({y}_{i},{\sigma }_{i})}{\int w({y}_{i},{\sigma }_{i})f({y}_{i},{\sigma }_{i})d{y}_{i}}$$

where $f({y}_{i},{\sigma }_{i})$ denotes the (unweighted) probability density function of the effect sizes. Note that if $w({y}_{i},{\sigma }_{i})=1$ for all ${y}_{i}$, the weighted density is the same as the density of the (equal-effect or random-effects) effect size model (Hedges & Vevea, 2005) and estimates of the selection model approach coincide with those of the effect size model. Maximum likelihood estimation is commonly applied to estimate *μ*, *τ*^2^, and the parameters in the weight function.

Selection model approaches mainly differ in how the selection model is specified. Hedges (1984) proposed the first selection model approach that assigned a weight of 1 to all statistically significant effect sizes and a weight of 0 to nonsignificant effect sizes. This method is comparable to *p-*uniform and *p-*curve except that maximum likelihood estimation is used in this selection model approach. Based on previous work of Iyengar and Greenhouse (1988a, b, Hedges (1992) formulated the 3PSM with a weight function that distinguishes statistically significant effect sizes (with a weight of 1) and nonsignificant effect sizes that are published at an unknown and to be estimated weight.

Multiple simulation studies have been conducted to study the performance of 3PSM and other selection model approaches as well as to compare these approaches to other publication bias methods. The overarching conclusion of these studies is that selection model approaches are among the best methods to correct for publication bias in a meta-analysis. However, Hong and Reed (2021) also emphasize that the performance of the methods depends on the used simulation design and metrics used for evaluating the methods. We summarize the results of previously conducted simulation studies in the OSM (Supplement 1).

## From *p*-uniform to *p*-uniform*

### *P*-uniform

*P*-uniform (van Aert et al., 2016; van Assen et al., 2015) uses the statistical principle that *p-*values are uniformly distributed at the true effect size. The method discards statistically nonsignificant effect sizes and only uses the significant effect sizes to correct for publication bias. Assumptions of the method are that a fixed true effect underlies the primary studies included in the meta-analysis and that all primary studies’ effect sizes that are statistically significant in the same direction have an equal probability of getting included in a meta-analysis. Statistical significance is taken into account – and hence there is corrected-for publication bias – by computing probabilities of observing an effect size or larger conditional on the effect size being statistically significant. The conditional probability of the *i*th study (${q}_{i}$) can be written as

2 $${q}_{i}=\frac{1-\Phi \left(\frac{{y}_{i}-\mu }{{\sigma }_{i}}\right)}{1-\Phi \left(\frac{{y}_{i}^{cv}-\mu }{{\sigma }_{i}}\right)}$$

where the numerator is the probability of observing an effect size at the true effect size larger than the effect size in the *i*th primary study and the denominator is the probability of observing a (statistically significant) effect size (i.e., larger than the critical value ${y}_{i}^{cv}$, which is the smallest statistically significant effect size given an α-level and ${\sigma }_{i}$). *P*-uniform can also be seen as a selection model approach with the equal-effect model as effect size model, and a selection model assuming equal weights for statistically significant effect sizes to get published.

*P*-uniform’s effect size estimate is equal to the value of *μ* where a statistic that is computed based on the ${q}_{i}$ equals its expected value assuming a uniform distribution. van Assen et al. (2015) proposed to use Fisher’s test (Fisher, 1925), $-\sum_{i=1}^{k}\mathrm{ln}({q}_{i})$, to estimate the effect size in *p-*uniform and draw statistical inference. van Assen et al. (2015) compared *p*-uniform with trim-and-fill (Duval & Tweedie, 2000a, 2000b) to correct for publication bias, and concluded that *p*-uniform outperformed trim-and-fill if publication bias exists and between-study variance in true effect size is absent or small. However, they also showed that overestimation of *p-*uniform increased as a function of the between-study variance in true effect sizes.

Another proposed estimator^2^ for estimating the effect size and computing the confidence interval with *p-*uniform is based on the distribution of the sum of independently uniformly distributed random variables, which is called the Irwin-Hall distribution (van Aert et al., 2016). van Aert et al. (2016) recommended using the estimator based on the Irwin-Hall distribution as the default estimator, mostly because the estimator based on the Irwin-Hall distribution is less susceptible to outlying effect sizes than the estimator using the Fisher’s test.

Three major drawbacks of *p*-uniform are that: (i) it is an inefficient estimator, because the methods only use statistically significant effect sizes; (ii) effect size estimates are positively biased if between-study variance in true effect sizes is present (Carter et al., 2019; McShane et al., 2016; van Aert et al., 2016); and (iii) the between-study variance is not estimated and tested. McShane et al. (2016) argued that an additional drawback of *p-*uniform is that the default estimation procedure is not maximum likelihood estimation. The next section introduces the generalization of *p*-uniform, *p*-uniform*, which addresses all these drawbacks.

### *P*-uniform*

*P*-uniform* is a selection model approach with the random-effects model as effect size model. The selection model assumes that the probability of publishing a statistically significant effect size as well as a nonsignificant effect size are constant, but these two probabilities may be different from each other. Hence, *p*-uniform* is a selection model approach with one cut-off at the critical value determining whether an effect size is statistically significant or not.

Maximum likelihood estimation is used in *p*-uniform*, where truncated densities are being used instead of the conditional probabilities in Eq. (2). Truncated densities (${q}_{i}^{M{L}^{*}}$) are computed for both the statistically significant and the nonsignificant effect sizes and are a function of both *μ* and ${\tau }^{2}$

3 $${q}_{i}^{M{L}^{*}}=\left\{\begin{array}{c}\frac{\frac{1}{\sqrt{{\sigma }_{i}^{2}+{\tau }^{2}}}\phi \left(\frac{{y}_{i}-\mu }{\sqrt{{\sigma }_{i}^{2}+{\tau }^{2}}}\right)}{1-\Phi \left(\frac{{y}_{i}^{cv}-\mu }{\sqrt{{\sigma }_{i}^{2}+{\tau }^{2}}}\right)}\, if\, {p}_{i}\le \alpha ,\\ \frac{\frac{1}{\sqrt{{\sigma }_{i}^{2}+{\tau }^{2}}}\phi \left(\frac{{y}_{i}-\mu }{\sqrt{{\sigma }_{i}^{2}+{\tau }^{2}}}\right)}{\Phi \left(\frac{{y}_{i}^{cv}-\mu }{\sqrt{{\sigma }_{i}^{2}+{\tau }^{2}}}\right)}\, if\, {p}_{i}>\alpha \end{array}\right.$$

where $\phi$ denotes the standard normal probability density function. The numerators of the truncated densities in Eq. (3) (i.e., the usual likelihoods) are weighted by the reciprocal of the probability of observing a (non)significant effect size given *μ*, ${\tau }^{2}$, and ${\sigma }_{i}^{2}$. An intuitive explanation of Eq. (3) is that the densities of statistically significant and nonsignificant effect sizes are computed given *μ* and ${\tau }^{2}$ conditional on an effect size being significant (top part of (3)) or not (bottom of (3)). As Eq. (3) does not contain a term for the probability of an effect size to be statistically (non)significant, publication bias (which is directly related to (non)significant studies getting published) is “conditioned away” or corrected for in *p*-uniform*. The likelihood function is the product of the ${q}_{i}^{M{L}^{*}}$ :

4 $$L(\mu ,{\tau }^{2})=\prod\limits_{i=1}^{k}{q}_{i}^{M{L}^{*}}$$

Equation (3) may at first glance not seem to be a generalization of Eq. (2) that is used in *p*-uniform. This is caused by the *q*_i_ in Eq. (2) being *conditional probabilities* and the ${q}_{i}^{M{L}^{*}}$ in Eq. (3) being *truncated densities*, because method of moments estimation is used by default in *p*-uniform, whereas *p*-uniform* is introduced above using maximum likelihood estimation. *P*-uniform* simplifies to *p*-uniform if maximum likelihood estimation is used in *p*-uniform and ${\tau }^{2}=0$. We also implemented *p-*uniform* with method of moments estimation using the Irwin-Hall distribution and the Fisher’s test as estimator. We describe the procedure for estimation with the method of moments estimators and the results of the simulation study using these estimators in the OSM (Supplement 2).

We compute 95% confidence intervals for *μ* and ${\tau }^{2}$ with *p-*uniform* using maximum likelihood estimation by inverting the likelihood-ratio test statistic. The likelihood-ratio test (Agresti, 2013; Pawitan, 2013) is used to test the null hypotheses *μ* = 0 and ${\tau }^{2}=0$ that compare the null model where either *μ* or ${\tau }^{2}$ is fixed to 0 with the alternative model where both parameters are estimated. Wald-based confidence intervals and hypothesis tests can also be conducted, but this is under the assumption that the log-likelihood around the maximum likelihood estimate is regular (Pawitan, 2013). Since *p-*uniform* is based on truncated densities, this assumption is likely not tenable and especially not when there are only a small number of primary studies in a meta-analysis. Hence, we implemented confidence intervals and hypothesis tests based on the likelihood-ratio test rather than on the Wald test.

## *P*-uniform* compared to 3PSM

3PSM requires that at least one study is observed in the intervals. In the most extreme case, with just one study in each of the two intervals, both 3PSM and *p*-uniform* only have a small bias for estimating the effect size but substantial bias for estimating the between-study variance (see Supplement 3 (OSM)). One study per interval technically also allows for estimation of the weights of 3PSM. However, it has been recommended that at least 10–15 studies are required in each interval to allow accurate estimation of the weights for more complex selection models with multiple steps (Hedges & Vevea, 1996, 2005; Vevea & Woods, 2005). Estimation of the average effect size and between-study variance in true effect sizes might still be quite accurate even if the weights are estimated imprecisely (Hedges & Veva, 1996).

There is no requirement in terms of number of effect sizes per interval for *p-*uniform*. This is the main difference between *p*-uniform* and 3PSM, because *p*-uniform* does not estimate the weight parameter. Hence, *p*-uniform* is a more parsimonious model than 3PSM and circumvents the issue of the potential imprecise estimation of the weight in 3PSM (Hedges & Vevea, 1996, 2005; Vevea & Woods, 2005). More parsimonious models are generally preferred over more complicated models if the performance of the models is comparable.

## Simulation study

This section describes the design and results of a simulation study to compare the statistical properties of the proposed *p*-uniform* method with the random-effects model, *p*-uniform, and 3PSM. The first goal of the simulation study is to examine the improvement of *p*-uniform* over *p*-uniform. The second goal is to compare the statistical properties of *p*-uniform* with those of the random-effects model and 3PSM.

### Method

Standardized mean differences were the effect size measure of interest using a two-independent groups design with a sample size of ${n}_{i}=50$ per group. We used Hedges’ *g* standardized mean difference rather than Cohen’s *d*, because a small positive bias in Cohen’s *d* is corrected for by Hedges’* g* (Hedges, 1981). The Hedges’ *g* effect size is obtained by multiplying Cohen’s *d* with the correction factor $J=\frac{\Gamma \left(\frac{df}{2}\right)}{\sqrt{\frac{df}{2}} \Gamma \left(\frac{df-1}{2}\right)}$ where Г refers to the gamma function and *df* to the degrees of freedom.

We started the simulation study by first sampling a true effect size ${\theta }_{i}$ for the *i*th primary study from *N*(*μ*,${\tau }^{2}$). Let ${\widetilde{n}}_{i}=\frac{{n}_{i}}{2}$ and ${g}_{i}$ being the observed Hedges’ *g* effect size in the *i*th study. The transformation ${J}^{-1}\sqrt{{\widetilde{n}}_{i}}{g}_{i}$ approximates a non-central *t-*distribution with *df* degrees of freedom and non-centrality parameter ${\theta }_{i}\sqrt{{\widetilde{n}}_{i}}$ (Hedges, 1981, 1983; Viechtbauer, 2005). Hedges’ *g* effect size ${g}_{i}$ was then obtained by sampling a *t*-value from this non-central *t*-distribution and dividing the sampled *t-*value by ${J}^{-1}\sqrt{{\widetilde{n}}_{i}}$. The unbiased estimate of the sampling variance of Hedges’ *g* (see Equation 26 in Viechtbauer, 2007a) was computed with $\frac{1}{{\widetilde{n}}_{i}}+\left(1-\frac{df\,-\,2}{df\,\times \,J{)}^{2}}\right){g}_{i}^{2}$. 

The effect size of the *i*th primary study was always included in the meta-analysis if it was statistically significant and positive. A right-tailed hypothesis test with α =.025 was used to resemble common practice of research in psychology where a two-tailed test with α =.05 is conducted and only effect sizes in the predicted direction are reported. Statistically nonsignificant effect sizes were included in the meta-analysis if a randomly drawn number from a uniform distribution ranging from zero to one was smaller than $1-pub$, where $1-pub$ represents the probability of a statistically nonsignificant effect size to be included in a meta-analysis with $pub=1$ referring to extreme publication bias (only statistically significant studies get published). This procedure for generating data of primary studies was repeated until *k* primary studies’ effect sizes were included in a meta-analysis.

A simulated meta-analysis may, by chance, include only statistically significant or only statistically nonsignificant effect sizes. This implies that there are no studies in one of the intervals of the selection model and 3PSM cannot be applied in this situation. These are often also challenging conditions for *p*-uniform*. The estimate of *p*-uniform* is, like the estimate of *p*-uniform (van Aert et al., 2016), expected to be (very) negative if there are only statistically significant effect sizes in a meta-analysis when some of these effect sizes have *p-*values slightly smaller than the α-level. A meta-analysis with only statistically significant effect sizes also makes it difficult for *p*-uniform* to estimate the between-study variance, as approximately the same fit is obtained with different (*µ,* ${\tau }^{2}$) pairs. Therefore, we also ran the simulation study where we randomly replaced one effect size from the meta-analysis with a nonsignificant one if the meta-analysis only contained significant effect sizes. In this simulation study, we also randomly replaced a statistically significant effect size with one nonsignificant effect size in case of only significant effect sizes in the meta-analysis.

The following variables were varied in the simulations: *μ*, *τ*, *k*, and *pub*. Three different levels were selected for *μ* (0; 0.2; 0.5) reflecting no, a small, and a medium effect (Cohen, 1988). The square root of the between-study variance in true effect sizes (*τ*) was 0, 0.163, or 0.346 representing *I*^2^-statistics equal to 0%, 40%, and 75% (zero, small-medium, large; Higgins, Thompson, Deeks, & Altman, 2003). The number of effect sizes in a meta-analysis (*k*) was equal to 10, 30, 60, and 120; 10 and 30 are close to the median (12) and mean (38.7) number of effect sizes in meta-analyses in psychology (van Erp et al., 2017), respectively. We also included 60 and 120 because previous research (Field & Gillett, 2010; Hedges & Vevea, 2005; Vevea & Woods, 2005) suggests that a large number of effect sizes in a meta-analysis are required in order for selection model approaches to perform well. Four different levels for *pub* were selected: 0, 0.5, 0.9, and 1. Combining the different levels of these variables resulted in 3 × 3 × 4 × 4 = 144 conditions. For each condition, 10,000 runs were conducted.^3^

*P-*uniform* and 3PSM were applied to each simulated meta-analysis. *P-*uniform* was implemented using maximum likelihood estimation and estimation based on the Irwin-Hall distribution and Fisher’s test (Fisher, 1925). The random-effects model was included to be able to compare methods that correct for publication bias with the method that is usually applied and does not correct for publication bias. We used the Paule-Mandel estimator (Paule & Mandel, 1982) to estimate the between-study variance in true effect sizes in the random-effects model, because this is one of the recommended estimators of the between-study variance (Langan, Higgins, & Simmonds, 2016; Veroniki et al., 2016). The outcome variables were the average, median, and standard deviation of the estimates, RMSE, and coverage probability and average width of the 95% confidence intervals for *μ* and *τ*.^4^

The simulation study was programmed in R (R Core Team, 2024) and the packages “metafor” (Viechtbauer, 2010), “weightr” (Coburn & Vevea, 2016), and “puniform” (van Aert, 2022) were used for applying the random-effects model, 3PSM, and *p*-uniform respectively. 3PSM was applied by using the implementation of “weightr” and “metafor,” because both implementations differ slightly. The implementation of 3PSM in “weightr” assigns the arbitrary weight of 0.01 in case it could not be estimated due to the absence of statistically nonsignificant effect sizes in a meta-analysis. Note that 3PSM cannot be fitted to a meta-analysis in the implementation of “metafor” if there are no statistically nonsignificant effect sizes in a meta-analysis. Furthermore, profile likelihood confidence intervals are implemented for ${\tau }^{2}$ in “metafor” whereas confidence intervals for ${\tau }^{2}$ are not implemented in “weightr.” Other R packages that were used to decrease the computing time of the simulations were the “parallel” package (R Core Team, 2024) for parallelizing the simulations and the “Rcpp” package (Eddelbuettel, 2013) for executing C++ functions. R code of the Monte-Carlo simulation study where a study is replaced in case of only statistically (non)significant effect sizes is available at https://osf.io/dyt4b?view_only=c17ce4ec24b748e7b2dff33dcd42942e, and R code of the simulation study without replacement is available at https://osf.io/f7ygu?view_only=c17ce4ec24b748e7b2dff33dcd42942e.

### Results

We do not present the results of the simulation study without replacement in case of only statistically significant or only nonsignificant effect sizes in a meta-analysis. None of the methods performed well in this situation as described in Supplement 4 (OSM). The overarching conclusion of this simulation study was that *p-*uniform* and 3PSM should not be applied in case of only statistically significant or nonsignificant effect sizes in a meta-analysis. The estimates of *p-*uniform, *p*-uniform*, and 3PSM as implemented in “weightr” were all severely biased in many conditions. 3PSM as implemented in “metafor” did not yield estimates in the vast majority of simulated meta-analyses, because there were often no statistically nonsignificant effect sizes in a meta-analysis.

In the remainder of this section, we therefore present the results of the simulation study where a study was replaced if there were initially only statistically significant or only nonsignificant effect sizes. We only present the results of *p-*uniform* with maximum likelihood estimation, because this estimator outperformed the estimators using Fisher’s test (Fisher, 1925) and the Irwin-Hall distribution. The width of the confidence intervals is not presented, because coverage probabilities often substantially deviated from the nominal coverage rate, thereby decreasing the usefulness of assessing the width of confidence intervals. The results of the Type-I error rate and statistical power are also not presented, because we wanted to primarily focus on the outcomes related to parameter estimation in the paper. Finally, we only present the results for *k* = 10 and 60 in this section, because these conditions already illustrate how the methods’ performances increase in *k*; the condition *k* = 120 was omitted because the methods’ performance in that condition was not remarkably different from that in *k* = 60. All results that are not presented in the paper are available online (see Supplement 1 (OSM)).

#### **Estimating***μ*

**Fig. 1:**
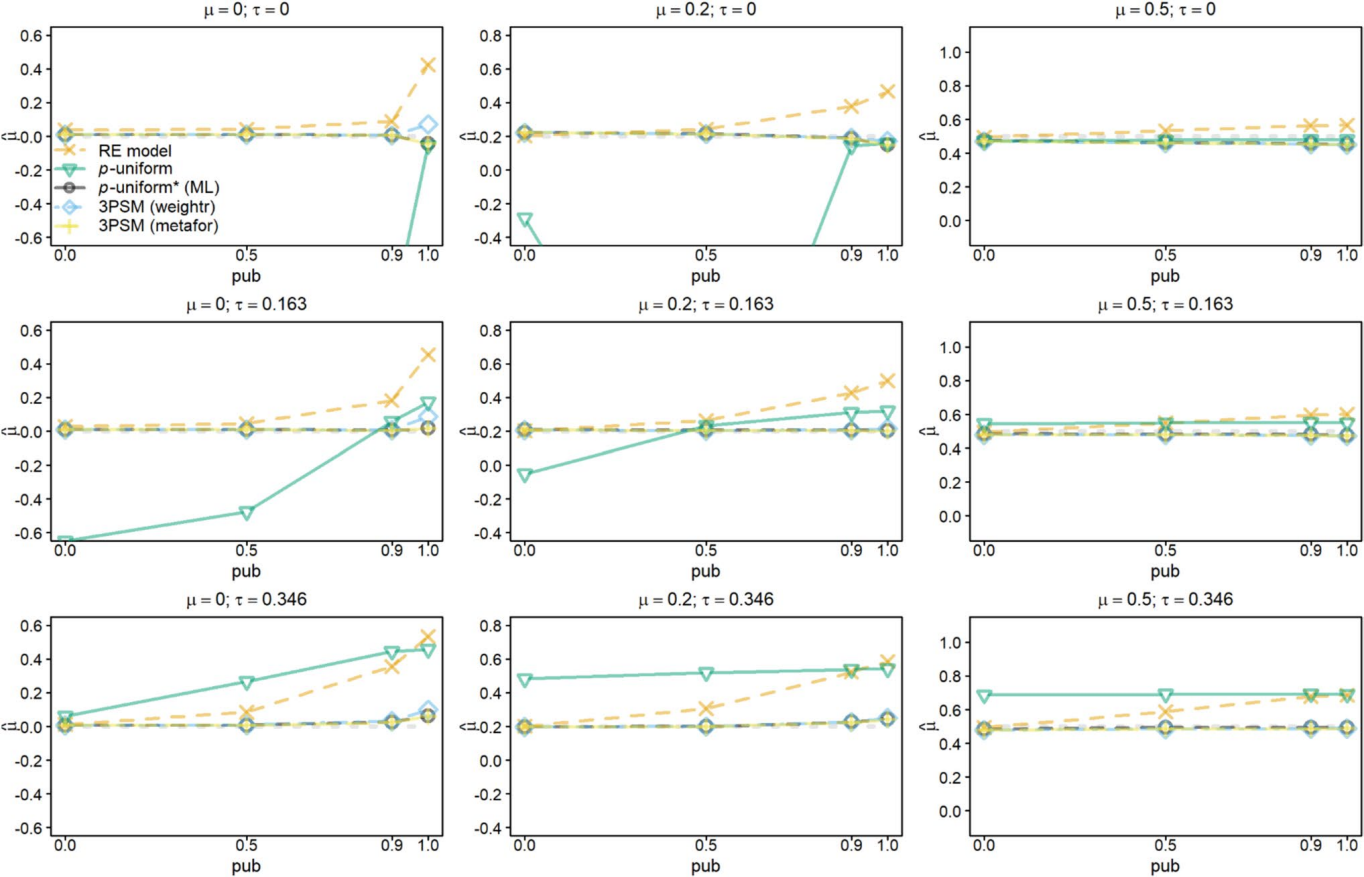
Average of the estimates of *μ* for the random-effects model (RE), *p-*uniform, *p*-uniform* using maximum likelihood estimation (ML), and the three-parameter selection model (3PSM) as implemented in the R packages “weightr” and “metafor.” The average of the estimates of *μ* is shown as a function of *μ*, *τ*, and the severity of publication bias (*pub*) with the number of primary studies’ observed effect sizes (*k*) equal to 10

**Fig. 2:**
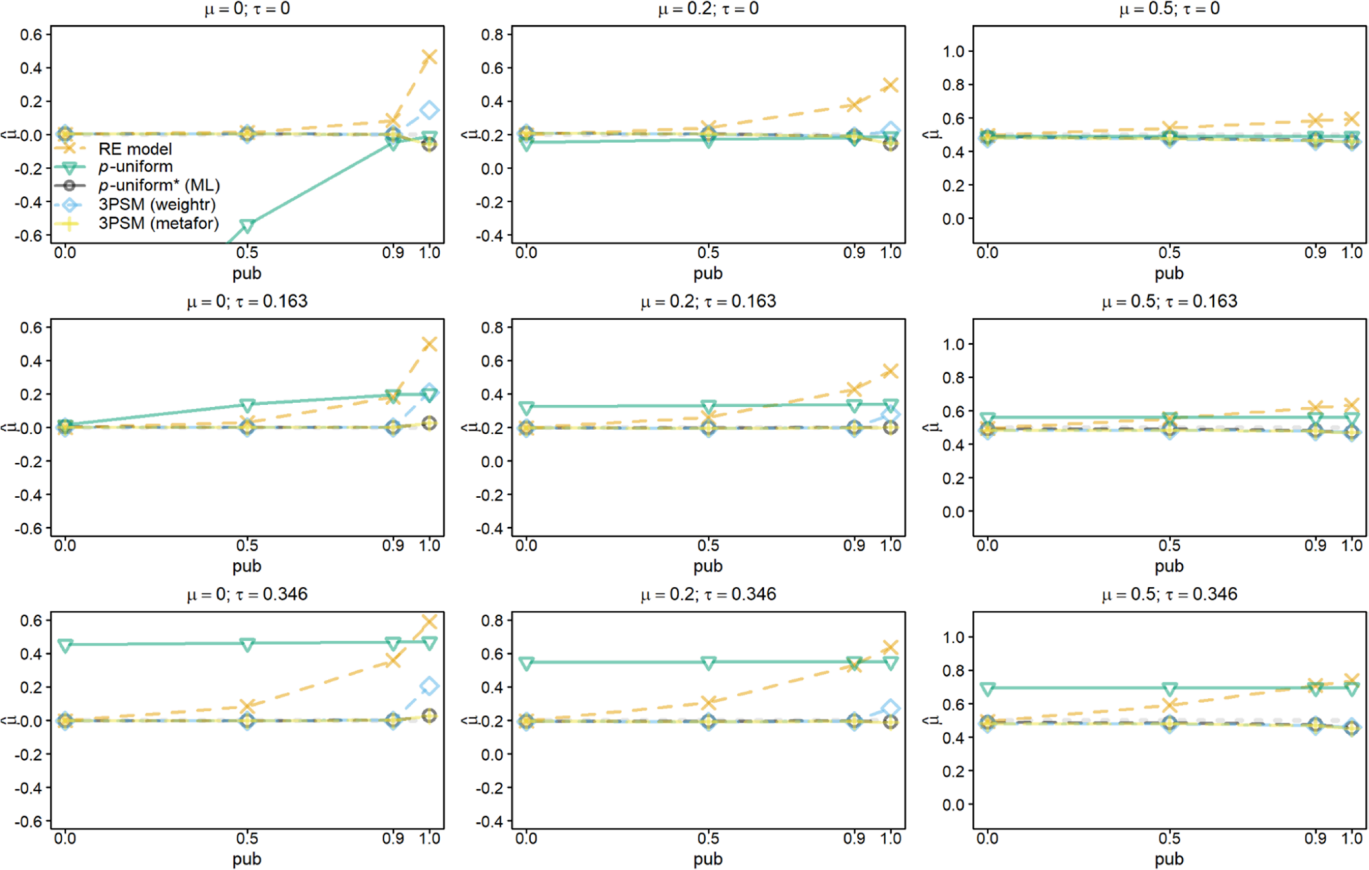
Average of the estimates of *μ* for the random-effects model (RE), *p-*uniform, *p*-uniform* using maximum likelihood estimation (ML), and the three-parameter selection model (3PSM) as implemented in the R packages “weightr” and “metafor.” The average of the estimates of *μ* is shown as a function of *μ*, *τ*, and the severity of publication bias (*pub*) with the number of primary studies’ observed effect sizes (*k*) equal to 60

Figures 1 and 2 show the average of the estimates of *μ* when *μ* (columns of the figures), *τ* (rows of the figures), and *pub* (*x*-axis of the figures) were varied for *k =* 10 and *k* = 60, respectively. All the figures are centered at the true effect size *μ* (dashed gray line) to facilitate comparability of the different subfigures as we varied *μ*. We first describe the results of *k* = 10 and then illustrate how the results change if *k* = 60.

Highlighting common issues with a lack of correction for publication bias, the random-effects model overestimated *μ* under publication bias and this overestimation decreased in *μ* and increased in *τ* and *pub*. *P*-uniform yielded a large negative bias if *pub* < 1 especially in conditions where only a small number of effect sizes per meta-analysis was statistically significant. This negative bias was caused by effect sizes with *p-*values close to the α-level. The performance of *p-*uniform* and both implementations of 3PSM was similar, resulting in overlapping lines and symbols in Fig. 1. The bias was negligible for all levels of *pub*. The bias of 3PSM as implemented in “weightr” was slightly larger than of *p*-uniform* and the implementation in “metafor” if *μ* = 0 (first column of figures) and *pub* = 1.

Bias of the random-effects model was unaffected by increasing the number of studies to 60 (see Fig. 2). Bias of *p*-uniform* and metafor’s implementation of 3PSM was also negligible for *k* = 60. The positive bias of the weightr’s implementation of 3PSM when *pub* = 1 increased for *μ* = 0 and was also present for *μ* = 0.2. The positive bias of the “weightr” implementation was a function of the number of studies in the meta-analysis, because the bias was larger for *k* = 120 compared to 60 (see Supplement 1 (OSM)).

#### **RMSE for estimating***μ*

**Fig. 3:**
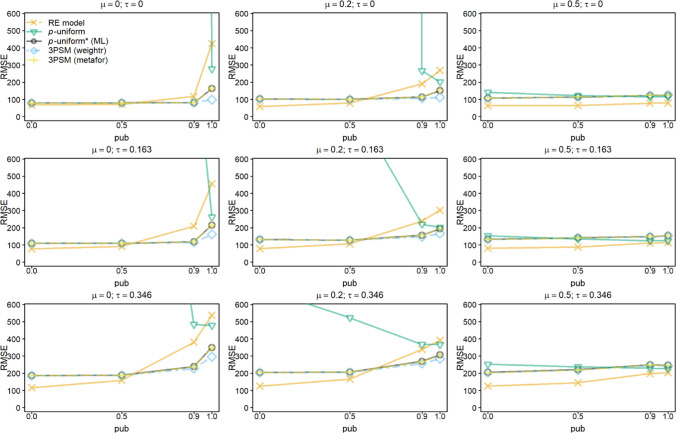
Root mean square error (RMSE) of estimating *μ* for the random-effects model (RE), *p-*uniform, *p*-uniform* using maximum likelihood estimation (ML), and the three-parameter selection model (3PSM) as implemented in the R packages “weightr” and “metafor.” The RMSE of estimating *μ* is shown as a function of *μ*, *τ*, and the severity of publication bias (*pub*) with the number of primary studies’ observed effect sizes (*k*) equal to 10. The RMSE was multiplied by 1,000 to facilitate the interpretation

**Fig. 4:**
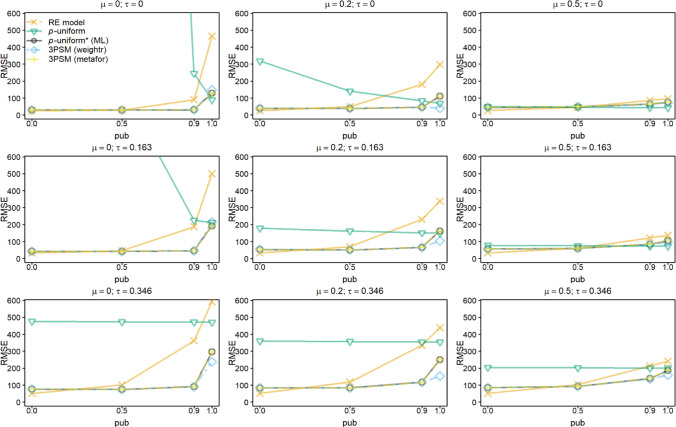
Root mean square error (RMSE) of estimating *μ* for the random-effects model (RE), *p-*uniform, *p*-uniform* using maximum likelihood estimation (ML), and the three-parameter selection model (3PSM) as implemented in the R packages “weightr” and “metafor.” The RMSE of estimating *μ* is shown as a function of *μ*, *τ*, and the severity of publication bias (*pub*) with the number of primary studies’ observed effect sizes (*k*) equal to 60. The RMSE was multiplied by 1,000 to facilitate the interpretation

Figures 3 and 4 show the RMSE for estimating *μ* for *k* = 10 and 60. The RMSE for the random-effects model followed the patterns observed for its bias; RMSE increased in publication bias and *τ*, and decreased in* μ*. Not only for *pub =* 0, but also for *pub =* 0.5, the random-effects model had a lower RMSE than the other methods. This was caused by the higher precision of the random-effects model since the bias of *p-*uniform* and both implementations of 3PSM was comparable to or smaller than of the random-effects model.

*P*-uniform’s RMSE was in the vast majority of conditions larger than that of *p*-uniform* and the two implementations of 3PSM. *P*-uniform was expected to outperform all other methods in the conditions with *τ* = 0 in combination with *pub* = 1, because there is no heterogeneity as assumed in *p*-uniform and there is extreme publication bias. However, its RMSE is larger than that of *p-*uniform* and the two implementations of 3PSM. This was caused by the single statistically nonsignificant effect size that replaced a significant effect size in these simulations. *P*-uniform was then based on only the nine statistically significant effect sizes, and this resulted in a higher RMSE than *p*-uniform* and the two implementations of 3PSM.

RMSE of *p*-uniform* and both implementations of 3PSM were comparable in all conditions except for *pub* = 1. If *pub* = 1, the implementation of 3PSM in “weightr” yielded a smaller RMSE if *μ* = 0 or 0.2. The reason for this is that the weight parameter in the 3PSM implementation of “weightr” is constrained to be at least 0.01, preventing extremely negative effect size estimates that may still occur for *p*-uniform* and “metafor.”

*P*-uniform benefitted most from increasing *k* to 60. It was among the methods with the smallest RMSE in the conditions with *τ* = 0 in combination with *pub* = 1. RMSE of the other methods that correct for publication bias were comparable. The RMSE of the 3PSM implementation in “weightr” was again sometimes lower than that of *p*-uniform* and the implementation of 3PSM in “metafor” due to constraining the weight parameter to 0.01 or larger.

#### **Coverage probability of confidence interval for*** μ*

A confidence interval for *μ* could always be computed for the random-effects model and *p-*uniform* but not with the two implementations of 3PSM. Confidence intervals of 3PSM could not be computed in at most 47.6% for the implementation in “weightr” and 26.8% for the implementation in “metafor” (condition* μ=0*,* τ* = 0, *k*=10, *pub* = 1). *P*-uniform’s confidence interval could, similarly to its effect size estimation, only be computed if a meta-analysis contained statistically significant effect sizes. The coverage probabilities shown in this section were determined based on all confidence intervals that could be computed for a particular method. This makes directly comparing the methods difficult since the number of confidence intervals that could be computed differed across methods. Furthermore, it is important to emphasize that differences in coverage probabilities between 3PSM and *p*-uniform* can be caused by the differences between the methods or by the fact a Wald-based confidence interval is computed by 3PSM and a profile likelihood confidence interval by *p*-uniform*.

Table 1 presents the coverage probability of the 95% confidence interval for *μ* if *k* = 10 and *k* = 60. Coverage probabilities between 0.9 and 0.975 are marked in bold and are deemed to be acceptable. Coverage probabilities of the random-effects model decreased as a function of *pub*. Coverage probabilities of all methods that correct for publication bias were close to the nominal coverage rate if *k* = 10 in combination with *τ* = 0. These results confirm that *p-*uniform yields exact confidence intervals if its assumptions are met. Nonzero values of *τ* in combination with the presence of publication bias resulted in under-coverage of all methods. This under-coverage was most severe if *pub* = 1 and performance of none of the methods was acceptable in this condition, with coverage probabilities being 0.000 for the random-effects model, 0.231 for *p-*uniform, 0.712 for *p*-uniform*, 0.832 for the “weightr” implementation of 3PSM, and 0.834 for the “metafor” implementation of 3PSM. It is remarkable that the coverage probabilities of *p*-uniform* were never larger than 0.975. This indicates that *p*-uniform* suffered more from under- than over-coverage. If *p*-uniform* had a too low coverage probability for a particular condition, the under-coverage always became worse if *τ* or *pub* was increased. The under-coverage of all methods became worse for *k* = 60 except for large between-study variance (*τ* = 0.346).

**Table 1:** Coverage probability of the confidence interval for *μ* computed with the random-effects model (RE), *p*-uniform, *p*-uniform* using maximum likelihood estimation (ML), and the three-parameter selection model (3PSM) as implemented in the R packages “weightr” and “metafor.” The coverage probabilities are shown as a function of *μ*, *τ*, the severity of publication bias (*pub*), and the number of primary studies’ observed effect sizes (k). Coverage probabilities between 0.9 and 0.975 are indicated in bold

		*k *= 10											
		*μ=0*				*μ* =0.2				*μ*=0.5			
	*pub*	0	0.5	0.9	1	0	0.5	0.9	1	0	0.5	0.9	1
$\tau$= 0	RE model	**0.960**	**0.957**	0.867	0.000	**0.972**	**0.920**	0.354	0.003	**0.960**	**0.965**	**0.926**	**0.924**
	*p-*uniform	**0.941**	**0.946**	**0.949**	**0.948**	**0.952**	**0.950**	**0.951**	**0.948**	**0.949**	**0.952**	**0.945**	**0.947**
	*p*-uniform* (ML)	**0.952**	**0.955**	**0.957**	**0.947**	**0.950**	**0.955**	**0.957**	**0.946**	**0.954**	**0.952**	**0.940**	**0.941**
	3PSM (weightr)	**0.961**	**0.966**	**0.965**	0.982	**0.956**	**0.955**	**0.962**	0.983	**0.957**	**0.968**	**0.973**	**0.973**
	3PSM (metafor)	**0.969**	**0.974**	**0.968**	0.986	**0.969**	**0.968**	**0.964**	0.978	**0.965**	**0.975**	**0.973**	0.977
$\tau$= 0.163	RE model	**0.967**	**0.952**	0.657	0.000	**0.966**	0.897	0.290	0.009	**0.959**	**0.945**	0.871	0.867
	*p-*uniform	0.896	0.888	0.870	0.802	0.893	0.886	0.848	0.824	**0.907**	0.897	0.893	0.893
	*p*-uniform* (ML)	**0.923**	**0.925**	**0.924**	0.858	**0.917**	**0.922**	**0.908**	0.871	**0.928**	**0.921**	**0.914**	**0.911**
	3PSM (weightr)	**0.946**	**0.945**	**0.946**	**0.957**	**0.927**	**0.928**	**0.949**	**0.967**	**0.940**	**0.944**	**0.948**	**0.948**
	3PSM (metafor)	**0.951**	**0.949**	**0.938**	**0.942**	**0.932**	**0.931**	**0.936**	**0.935**	**0.944**	**0.947**	**0.952**	**0.956**
$\tau$= 0.346	RE model	**0.972**	**0.917**	0.353	0.003	**0.958**	0.875	0.251	0.030	**0.956**	**0.915**	0.740	0.704
	*p*-uniform	0.657	0.607	0.371	0.231	0.634	0.555	0.369	0.316	0.663	0.601	0.567	0.564
	*p*-uniform* (ML)	**0.901**	**0.904**	0.867	0.712	0.882	0.895	0.806	0.734	**0.902**	0.872	0.834	0.827
	3PSM (weightr)	**0.919**	**0.920**	**0.904**	0.832	0.892	**0.902**	0.870	0.844	**0.916**	**0.904**	0.888	0.882
	3PSM (metafor)	**0.920**	**0.920**	0.896	0.835	0.894	**0.902**	0.863	0.834	**0.917**	**0.905**	0.890	0.886
		*k* = 60											
		*μ=0*				*μ* =0.2				*μ*=0.5			
	*pub*	0	0.5	0.9	1	0	0.5	0.9	1	0	0.5	0.9	1
$\tau$= 0	RE model	**0.958**	**0.932**	0.299	0.000	**0.950**	0.707	0.000	0.000	**0.947**	0.665	0.012	0.000
	*p-*uniform	**0.951**	**0.952**	**0.951**	**0.951**	**0.951**	**0.949**	**0.946**	**0.949**	**0.947**	**0.948**	**0.946**	**0.947**
	*p*-uniform* (ML)	**0.952**	**0.951**	**0.953**	0.837	**0.943**	**0.951**	**0.952**	0.827	**0.944**	**0.925**	0.862	0.832
	3PSM (weightr)	**0.968**	**0.966**	**0.958**	0.160	0.980	**0.968**	**0.960**	**0.955**	**0.959**	0.977	0.998	0.999
	3PSM (metafor)	**0.962**	**0.960**	**0.956**	0.998	**0.971**	**0.961**	**0.951**	0.996	**0.955**	**0.962**	0.989	0.994
$\tau$= 0.163	RE model	**0.953**	0.876	0.012	0.000	**0.951**	0.613	0.000	0.000	**0.950**	0.618	0.004	0.000
	*p-*uniform	0.871	0.827	0.580	0.230	0.788	0.687	0.457	0.329	0.760	0.714	0.673	0.670
	*p*-uniform* (ML)	**0.922**	**0.932**	**0.946**	0.503	**0.907**	**0.939**	**0.912**	0.547	**0.938**	**0.915**	0.810	0.753
	3PSM (weightr)	**0.949**	**0.947**	**0.948**	0.840	**0.932**	**0.942**	**0.944**	**0.972**	**0.952**	**0.962**	**0.966**	**0.968**
	3PSM (metafor)	**0.950**	**0.947**	**0.948**	**0.943**	**0.934**	**0.942**	**0.945**	**0.947**	**0.952**	**0.962**	**0.963**	**0.965**
$\tau$= 0.346	RE model	**0.951**	0.684	0.000	0.000	**0.951**	0.504	0.000	0.000	**0.950**	0.544	0.000	0.000
	*p*-uniform	0.238	0.076	0.001	0.000	0.083	0.018	0.000	0.000	0.086	0.043	0.016	0.011
	*p*-uniform* (ML)	**0.911**	**0.932**	**0.931**	0.500	**0.923**	**0.940**	0.865	0.542	**0.936**	**0.915**	0.775	0.680
	3PSM (weightr)	**0.940**	**0.945**	**0.942**	0.776	**0.937**	**0.940**	**0.938**	0.863	**0.945**	**0.951**	**0.944**	**0.928**
	3PSM (metafor)	**0.940**	**0.945**	**0.942**	0.856	**0.937**	**0.940**	**0.938**	0.877	**0.945**	**0.951**	**0.944**	**0.928**

The coverage probabilities for *μ* = 0 shown in Table 1 can also be used to draw conclusions about the Type-I error rate of the test of no effect. Adequate Type-I error control with a probability of α = 0.05 for making a Type-I error would imply that the coverage rate is close to 0.95. As expected, for *k* = 10 and *τ* = 0 the Type-I error rate of both *p*-uniform and *p*-uniform* was close to the nominal Type-I error rate. Also as expected, *p*-uniform’s Type-I error rate was substantially higher than that of *p*-uniform* if between-study variance was present in the meta-analysis. This indicates that *p*-uniform* is indeed an improvement over *p*-uniform if between-study variance is present in a meta-analysis. However, *p*-uniform*’s Type-I error rate was still inflated, similar to that of 3PSM implementations of “metafor” and “weightr,” in case of *τ* = 0.346 and *pub* = 1, but the inflation was less severe for the implementation in “weightr” (0.168) and “metafor” (0.165) compared to *p*-uniform* (0.288). For *k* = 60, the Type-I error rate of the methods was generally larger than for *k* = 10. If *τ* = 0.346 and *pub* = 1, the Type-I error rate of the random-effects model and *p*-uniform was 1 and of *p*-uniform*, 3PSM implemented with “weightr” and “metafor” the Type-I error rate was 0.500, 0.224, and 0.144, respectively.

#### **Estimating***τ*

**Fig. 5:**
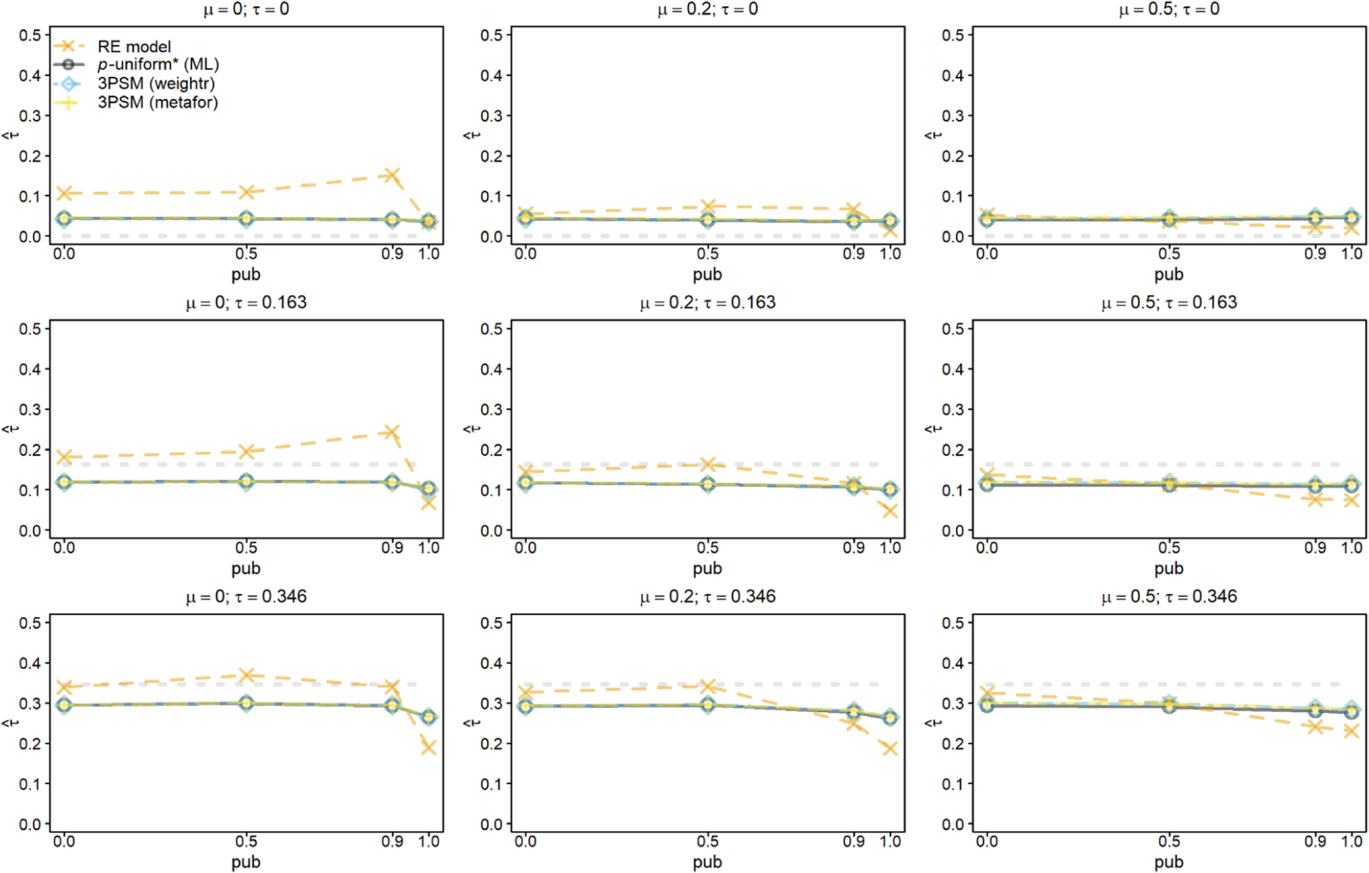
Average of the estimates of *τ* for the random-effects model (RE), *p*-uniform* using maximum likelihood estimation (ML), and the three-parameter selection model (3PSM) as implemented in the R packages “weightr” and “metafor.” The average of the estimates of *τ* is shown as a function of *μ*, *τ*, and the severity of publication bias (*pub*) with the number of primary studies’ observed effect sizes (*k*) equal to 10

**Fig. 6:**
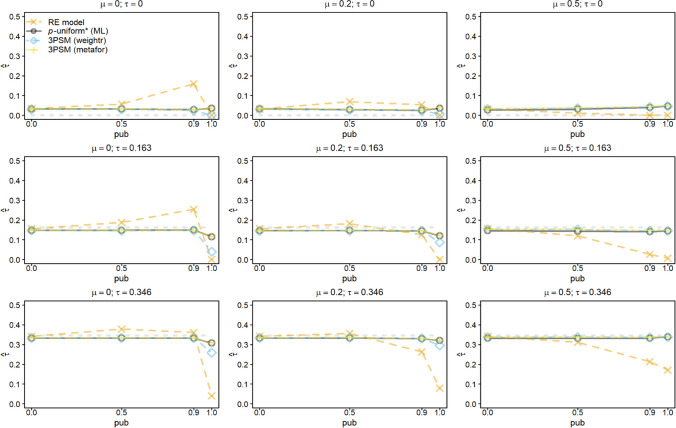
Average of the estimates of *τ* for the random-effects model (RE), *p*-uniform* using maximum likelihood estimation (ML), and the three-parameter selection model (3PSM) as implemented in the R packages “weightr” and “metafor.” The average of the estimates of *τ* is shown as a function of *μ*, *τ*, and the severity of publication bias (*pub*) with the number of primary studies’ observed effect sizes (*k*) equal to 60

Figures 5 and 6 show the average estimates of *τ* for *k* = 10 and 60, respectively. Note that *p-*uniform does not estimate *τ*. All methods were positively biased if *τ* = 0 in combination with *k* = 10, and the random-effects model yielded the least biased estimator for these conditions if *pub* = 1. The random-effects model yielded sometimes a larger bias than the other methods if *τ* = 0 in combination with *pub* = 0. This was caused by replacing a statistically nonsignificant effect size with a significant effect size in the simulations if there were only nonsignificant effect sizes in a meta-analysis. *P*-uniform* and the two implementations of 3PSM yielded comparable results. The methods were negatively biased *i*f *τ* > 0. Bias of the random-effects model was generally less than of the methods that correct for publication bias if *pub* < 0.9. Increasing *k* to 60 did especially decrease the bias of *p*-uniform* and 3PSM as implemented in “metafor.” The implementation of 3PSM in “weightr” was more negatively biased when than the other methods to correct for publication bias if *τ* > 0 in combination with *pub* = 1. This was again caused by the restricting the weight parameter to 0.01 or larger.

#### **RMSE for estimating***τ*

**Fig. 7:**
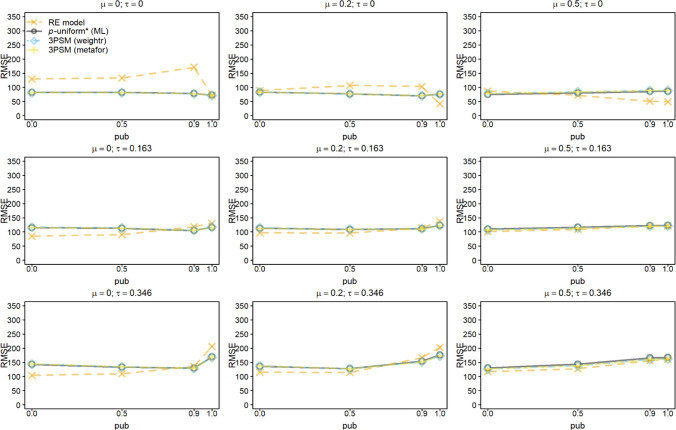
Root mean square error (RMSE) of estimating *τ* for the random-effects model (RE), *p*-uniform* using maximum likelihood estimation (ML), and the three-parameter selection model (3PSM) as implemented in the R packages “weightr” and “metafor.” The RMSE of estimating *τ* is shown as a function of *μ*, *τ*, and the severity of publication bias (*pub*) with the number of primary studies’ observed effect sizes (*k*) equal to 10. The RMSE was multiplied by 1,000 to facilitate the interpretation

**Fig. 8:**
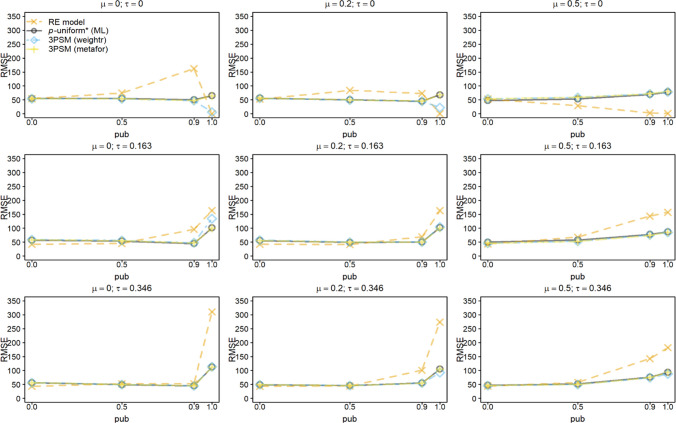
Root mean square error (RMSE) of estimating *τ* for the random-effects model (RE), *p*-uniform* using maximum likelihood estimation (ML), and the three-parameter selection model (3PSM) as implemented in the R packages “weightr” and “metafor.” The RMSE of estimating *τ* is shown as a function of* μ*, *τ*, and the severity of publication bias (*pub*) with the number of primary studies’ observed effect sizes (*k*) equal to 60. The RMSE was multiplied by 1,000 to facilitate the interpretation

Figures 7 and 8 present the RMSE for estimating *τ*. For *k* = 10 (Fig. 7), the RMSE of the random-effects model was higher than of the other methods for the condition *τ =* 0 in combination with *pub* = 0. This was caused by the replacement procedure that was used in these simulations. The random-effects model had a smaller RMSE than the other methods if *τ =* 0 in combination with *μ* > 0 and *pub* = 1. RMSE of all methods were comparable if *τ* > 0. For *k* = 60, RMSE of especially the methods that corrected for publication bias decreased compared to *k* = 10.

#### **Coverage probability of confidence interval for***τ*

Note that the implementation of 3PSM in “weightr” does not compute confidence intervals for *τ*. Table 2 presents the coverage probabilities of the random-effects model, *p-*uniform*, and 3PSM as implemented in “metafor.” Coverage probabilities between 0.9 and 0.975 are marked in bold and are evaluated as acceptable. For *k* = 10, the random-effects model yielded accurate coverage if *μ* = 0.5 and this was also the case if publication bias was present. Coverage probabilities of *p*-uniform* were especially too low if *pub* ≥ 0.9 and *τ* = 0.346. For example, the coverage probability of *p-*uniform* was 0.769 for the condition *μ* = 0 in combination with *pub* = 1 and *τ* = 0.346. The implementation of 3PSM in “metafor” yielded overcoverage for *τ* < 0.346 that could be as high as 0.993. Acceptable coverage of the 3PSM implementation in “metafor” was observed if *τ* = 0.346. Increasing the number of studies to *k* = 60 resulted in more conditions with acceptable coverage probabilities for *p*-uniform* and the implementation of 3PSM in “metafor” and less conditions with acceptable coverage of the random-effects model.

**Table 2:** Coverage probability of the confidence interval for ${\uptau }^{2}$ computed with the random-effects model (RE), *p*-uniform* using maximum likelihood estimation (ML), and the three-parameter selection model (3PSM) as implemented in the R package “metafor.” The coverage probabilities are shown as a function of *μ*, τ, the severity of publication bias (*pub*), and the number of primary studies’ observed effect sizes (*k*). Coverage probabilities between 0.9 and 0.975 are indicated in bold

		*k* = 10											
		*mμ=*0				*mμ=*0.2				*mμ=*0.5			
	*pub*	0	0.5	0.9	1	0	0.5	0.9	1	0	0.5	0.9	1
$\tau$= 0	RE model	**0.931**	**0.922**	0.775	0.824	**0.966**	**0.949**	**0.910**	0.758	**0.956**	**0.951**	**0.941**	**0.935**
	*p*-uniform* (ML)	0.983	0.982	0.985	**0.969**	**0.971**	0.982	0.985	**0.964**	0.984	**0.972**	**0.964**	**0.963**
	3PSM (metafor)	0.991	0.991	0.990	0.991	0.990	0.993	0.991	0.992	0.991	0.988	0.991	0.991
$\tau$= 0.163	RE model	**0.964**	**0.939**	0.818	0.778	**0.959**	**0.947**	0.894	0.750	**0.957**	**0.942**	**0.923**	**0.920**
	*p*-uniform* (ML)	**0.971**	**0.971**	0.976	**0.962**	**0.972**	0.980	0.979	**0.961**	0.984	**0.972**	**0.964**	**0.962**
	3PSM (metafor)	0.988	0.986	0.978	0.991	0.988	0.987	0.989	0.991	0.990	0.988	0.992	0.991
$\tau$= 0.346	RE model	**0.968**	**0.946**	**0.903**	0.752	**0.956**	**0.950**	0.863	0.771	**0.952**	**0.945**	**0.904**	**0.900**
	*p*-uniform* (ML)	**0.902**	**0.915**	0.893	0.769	**0.903**	**0.914**	0.842	0.781	**0.911**	0.892	0.845	0.847
	3PSM (metafor)	**0.930**	**0.931**	**0.925**	**0.922**	**0.927**	**0.927**	**0.927**	**0.931**	**0.933**	**0.943**	**0.941**	**0.945**
		*k* = 60											
		*μ=*0				*μ=*0.2				*μ=*0.5			
	*pub*	0	0.5	0.9	1	0	0.5	0.9	1	0	0.5	0.9	1
$\tau$= 0	RE model	**0.965**	**0.917**	0.159	0.000	**0.953**	0.887	0.890	0.000	**0.953**	0.845	0.246	0.099
	*p*-uniform* (ML)	0.980	0.981	0.988	0.867	**0.968**	0.979	0.986	0.862	0.986	**0.973**	**0.908**	0.874
	3PSM (metafor)	0.988	0.986	0.991	0.987	0.987	0.988	0.989	0.990	0.984	0.987	0.988	0.986
$\tau$= 0.163	RE model	**0.952**	0.876	0.212	0.000	**0.950**	**0.915**	0.836	0.000	**0.953**	0.860	0.217	0.061
	*p*-uniform* (ML)	**0.926**	**0.934**	**0.943**	0.484	**0.912**	**0.934**	**0.904**	0.498	**0.931**	0.899	0.781	0.710
	3PSM (metafor)	**0.944**	**0.944**	**0.937**	0.984	**0.941**	**0.942**	**0.939**	0.985	**0.952**	**0.955**	**0.964**	**0.968**
$\tau$= 0.346	RE model	**0.955**	0.888	0.895	0.000	**0.952**	**0.942**	0.556	0.001	**0.949**	0.875	0.200	0.030
	*p*-uniform* (ML)	**0.915**	**0.937**	**0.927**	0.502	**0.927**	**0.937**	0.865	0.547	**0.934**	**0.911**	0.768	0.668
	3PSM (metafor)	**0.949**	**0.949**	**0.939**	**0.942**	**0.946**	**0.940**	**0.939**	**0.954**	**0.947**	**0.951**	**0.954**	**0.956**

## Application

We applied random-effects meta-analysis, *p-*uniform, *p*-uniform*, and 3PSM using the implementations in the R packages “weightr” and “metafor” to two published meta-analyses in this section that differed in the amount of estimated between-study variance. The meta-analysis by Rabelo, Keller, Pilati, and Wicherts (2015) consists of 25 Hedges’ *g* standardized mean differences on whether participant’s judgment of importance of, for instance, morality-related outcomes changes if they are holding a heavy- or lightweight object. The total sample sizes of the primary studies ranged from 30 to 100 (mean = 61.12, median = 60). All effect sizes were positive and 21 (84%) effect sizes were statistically significant based on a two-tailed test with α =.05.

The methods were also applied to the meta-analysis by Bangert-Drowns, Hurley, and Wilkinson (2004). This meta-analysis consisted of 48 Hedges’ *g* standardized mean differences on the effect of writing-to-learn interventions on academic achievement. That is, students’ academic achievement in these primary studies was compared between an experimental group where there was an explicit focus on learning by writing and a control group where traditional teaching methods were used. Sampling variances of the Hedges’ *g* effect sizes were computed assuming equal sample sizes in both groups. The total sample sizes ranged from 16 to 542 (mean = 116.2, median = 67.5). Based on a two-tailed test with α =.05, 14 effect sizes were significantly larger than zero and one effect size was significantly smaller than zero. R code of applying the methods to these to meta-analysis is available at https://osf.io/9k3bp?view_only=c17ce4ec24b748e7b2dff33dcd42942e.

Table 3 shows the results of applying the methods to the meta-analysis of Rabelo et al. (2015) (first five rows) and Bangert-Drowns et al. (2004) (last five rows). The average effect size estimate of Rabelo et al. (2015) was substantially smaller when correcting for publication bias with *p*-uniform (−0.179), *p*-uniform* (0.244), and both implementations of 3PSM (0.254) when compared with the estimate not corrected for bias (0.571). The null hypothesis of no effect was rejected with all methods except for *p-*uniform. The between-study variance was estimated as zero by all methods that estimated this parameter and hence none of these methods rejected the null hypothesis of no heterogeneity. These results suggest that the effect of holding a heavy- or lightweight object on judgment of importance is closer to zero with all methods that correct for publication bias and approximately halves when *p-*uniform* and 3PSM were applied.

**Table 3:** Results of applying the random-effects meta-analysis model (RE), *p*-uniform, *p*-uniform* using maximum likelihood (ML) estimation, and 3PSM as implemented in the R package “weightr” and “metafor” to the data of the meta-analysis Rabelo et al. (2015) (first five rows) and Bangert-Drowns et al. (2004) (last five rows). Between-study variance in the random-effects meta-analysis model was estimated using the Paule-Mandel estimator

	*µ* (SE)	(95% CI *µ*)	H_0_: *µ* = 0	${\uptau }^{2}$(SE)	(95% CI ${\uptau }^{2}$)	H_0_: ${\uptau }^{2}$ = 0
Rabelo et al. (2015)						
RE model	0.571 (0.023)	(0.524; 0.618)	*t* = 25.036, *p <*.0001	0.000 (0.020)	(0.000; 0.000)	*Q = *4.553,* p = *1.000
* p*-uniform	−0.149 (-)	(−0.628; 0.186)	*L*_0_ = 0.802, *p* =.789	-	-	-
* p-*uniform* (ML)	0.244 (-)	(0.038; 0.436)	*L*_0_ = 5.327, *p* =.021	0.000 (-)	(0.000; 0.019)	*L*_het_ = 0.000, *p* =.500
3PSM (weightr)	0.254 (0.018)	(0.220; 0.289)	*z = *14.402, *p <*.0001	0.000 (-)	-	-
3PSM (metafor)	0.255 (0.100)	(0.060; 0.450)	*z = *2.560, *p <*.0001	0.000 (-)	(0.000; 0.019)	LRT = 0.000, *p* = 1.000
Bangert-Drowns et al. (2004)						
RE model	0.228 (0.051)	(0.126; 0.330)	*t* = 4.511, *p <*.0001	0.069 (0.025)	(0.027; 0.153)	*Q* = 107.106, *p <*.0001
* p*-uniform	0.245 (-)	(−0.236; 0.531)	*L*_0_ = −1.140, *p* =.127	-	-	-
* p-*uniform* (ML)	0.179 (-)	(0.065; 0.334)	*L*_0_ = 10.224,* p* =.001	0.027 (-)	(0.004; 0.079)	*L*_het_ = 6.311, *p* =.006
3PSM (weightr)	0.148 (0.073)	(0.004; 0.291)	*z = *2.020, *p* =.043	0.028, (0.024)	-	-
3PSM (metafor)	0.148 (0.073)	(0.004; 0.291)	*z = *2.018, *p* =.044	0.028 (-)	(0.000; 0.095)	LRT = 2.250, *p* =.134

*CI* = confidence interval, *LRT* = likelihood-ratio test. A dash indicates that a method does not return a particular result

 The correction for publication bias was smaller in the meta-analysis of Bangert-Drowns et al. (2004); the average effect size estimate of random-effects meta-analysis (0.228) was only slightly larger than estimates obtained with *p-*uniform* (0.179) and both implementations of 3PSM (0.148). *P*-uniform’s estimate (0.245) was larger than the estimate of random-effects meta-analysis, which was likely caused by *p-*uniform overestimating the average effect size due to heterogeneity in true effect sizes. The null hypothesis of no effect was rejected by all methods except for *p*-uniform. The estimated between-study variance by *p*-uniform* (0.027) and both implementations of 3PSM (0.028) was smaller than estimated in the random-effects meta-analysis (0.069), and the null hypothesis of no heterogeneity was no longer rejected with the implementation of 3PSM in “metafor.” To conclude, correcting for potential publication bias reduced the average effect size in the meta-analysis of Bangert-Drowns et al. (2004) when *p*-uniform* and 3PSM were applied. However, there was still evidence for the presence of the effect of writing-to-learn interventions on academic achievement when *p-*uniform* and 3PSM were applied.

## Discussion

Publication bias distorts the results of meta-analyses yielding overestimated effect sizes. Multiple methods were developed to correct for publication bias in a meta-analysis, and selection model approaches are seen as the state-of-the-art methods (McShane et al., 2016). *P*-uniform (van Aert et al., 2016; van Assen et al., 2015) can also be seen as a selection model approach, and we generalized and improved *p*-uniform to *p*-uniform*. *P*-uniform* does not only use statistically significant primary studies’ effect sizes for estimation as with *p*-uniform, it also uses the nonsignificant effect sizes, and results in three major improvements of *p*-uniform* over *p*-uniform: (i) it makes *p*-uniform* a more efficient estimator than *p*-uniform, (ii) overestimation of effect size by *p*-uniform in case of between-study variance in true effect is eliminated, and (iii) it enables estimation and testing for the presence of the between-study variance in true effect sizes.

The aim of this paper was to introduce *p*-uniform* and compare the statistical properties of the method with those of *p-*uniform, 3PSM, and the random-effects model that is commonly used but does not correct for publication bias. The simulation study confirmed previous research that the random-effects model yields overestimated average effect size and unpredictable bias in the between-study variances in true effect sizes (Augusteijn et al., 2019; Jackson, 2006, 2007) if publication bias is present. The simulation study also showed that *p*-uniform* is an improvement over *p-*uniform if between-study variance is present. Statistical properties of *p*-uniform* and 3PSM were generally comparable, but these were not acceptable in case of extreme publication bias with only statistically significant primary studies’ effect sizes in a meta-analysis. However, previous research (van Aert et al., 2016; van Assen et al., 2015) showed that *p-*uniform can be used in such a situation if heterogeneity in true effect sizes is zero or small.

Given that *p*-uniform* was shown to be a major improvement over *p*-uniform if between-study variance is present and *p-*uniform* showed comparable statistical properties to 3PSM, *p-*uniform* is a viable method to correct for publication bias. *P*-uniform* has the advantage over 3PSM that the weight in the selection model does not need to be estimated. These weights are known to be imprecisely estimated in more complex selection models than the selection model of 3PSM where multiple weights need to be estimated (Hedges & Vevea, 1996, 2005; Vevea & Woods, 2005). *P*-uniform* only assumes that these weights are the same for the statistically significant and the same for the nonsignificant primary studies’ effect sizes, without requiring estimation of the weights. This makes *p-*uniform* a more parsimonious model than 3PSM with similar statistical properties as shown in our simulation studies.

We provide recommendations for meta-analysts in practice based on the results of our simulation study. Foremost, we recommend not solely relying on the traditional meta-analysis models if publication bias may have affected the meta-analysis. This is in agreement with the Meta-Analytic Reporting Standards (MARS; Appelbaum et al., 2018) that recommends assessing the impact of publication bias in any meta-analysis. We advise researchers to use so-called triangulation where researchers do not rely on one particular publication bias method but use multiple publication bias methods that are known to have good statistical properties for the characteristics of the meta-analysis (Carter et al., 2019; Coburn & Vevea, 2015; Kepes et al., 2012). *P*-uniform* and 3PSM may be considered to be included in the triangulation given the adequate statistical properties in many conditions of our simulation studies. If the publication bias methods applied to a meta-analysis reach similar conclusions, the meta-analyst can be more confident in their results and conclusions based upon these results. In case of diverging conclusions by the methods, the interpretation of the results must be done with caution. Another novel approach to triangulation is Robust Bayesian Model Averaging (RoBMA; Bartoš et al., 2023), which includes multiple publication bias methods and computes pooled estimates by weighting the results of the methods with their posterior model probability.

Importantly, we do not recommend applying 3PSM using the implementation in “weightr” if there are only statistically significant effect sizes in a meta-analysis. The weight parameter is then fixed to 0.01 and this is not evidence-based. Moreover, 0.01 seems unrealistically small, implying that 99% of the statistically nonsignificant effect sizes end up in the file drawer. We do also not recommend using *p-*uniform* if the meta-analysis only contains statistically significant effect sizes caused by publication bias. Although, *p*-uniform* can technically, in contrast to 3PSM, be applied, our simulation study showed bad performance in this condition (see Supplement 4 (OSM)). If the between-study variance is expected to be zero or small, *p*-uniform can then better be applied since it provides estimates close to the true effect size and exact confidence intervals in these situations. However, we also suspect good performance of *p*-uniform* in this condition when all statistically significant effect sizes are accompanied by very small *p*-values (say <.001), suggesting that these significant effects are not caused by publication bias but by high power of the primary studies.

*P*-uniform* can be easily applied by meta-analysts using the function “puni_star()” in the R package “puniform.” Users can currently analyze primary studies based on a two-sample or one-sample *t*-test and correlation coefficient or can supply the function directly with standardized effect sizes. Meta-analysts who are not familiar with R can also apply *p*-uniform* to their data via an easy-to-use web application (https://rvanaert.shinyapps.io/p-uniformstar/). This web application can be applied if the primary study’s effect size measure is a one-sample mean, two-independent means or correlation coefficient. 3PSM can be applied using the “weightr” package (Coburn & Vevea, 2016), the corresponding web application (https://vevealab.shinyapps.io/WeightFunctionModel/), or the “metafor” package (Viechtbauer, 2010).

A limitation of *p*-uniform* and 3PSM is that the probability of publishing a statistically nonsignificant primary study’s effect size is assumed to be the same for all nonsignificant effect sizes in a meta-analysis. This assumption may be violated in practice causing biased estimates of the methods (for a discussion about this assumption, see Simonsohn, Simmons, & Nelson, 2017, December 20). To counteract a violation of this assumption, 3PSM can be extended to allow for more than two intervals of the selection model. *P-*uniform*’s selection model can, in principle, also be extended to allow for more than two intervals. For example, researchers may expect that the probability of publishing a primary study’s effect size with a marginally significant *p-*value (i.e., *p-*value between 0.05 and 0.1) is different from publishing a statistically nonsignificant effect size with a *p-*value larger than 0.1. This can be incorporated in *p*-uniform* where the truncated densities are computed differently for these three intervals. That is, the denominators of the truncated density in Eq. (3) for an effect size with a *p*-value smaller than 0.05, between 0.05 and 0.1, and larger than 0.1 equal the probability of observing a *p*-value in this interval given *µ* and *τ*. However, information needs to be available to select appropriate thresholds for these intervals. We simulated data in this study that exactly matched the selection models of *p-*uniform* and 3PSM, so future research may study to what extent statistical properties of the methods deteriorate if the selection model for simulating data differs from the methods’ selection model. Future research may also study the effect of publication bias on the meta-analytic results if covariates are included in a meta-analysis model.

Another limitation of *p-*uniform* and 3PSM, and in fact all methods of meta-analysis, is that their results will be distorted if *p-*hacking (also known as questionable research practices or researcher degrees of freedom, see Simmons, Nelson, & Simonsohn, 2011; Wicherts et al., 2016) are used in the primary studies that are included in a meta-analysis. An opportunity for future research is to examine whether existing publication bias methods can be adapted to be not biased in case of *p-*hacking. This is challenging, because there are different types of *p*-hacking that often differ in how they impact, for example, the effect size and *p-*value. An approach using a meta-regression model was developed (van Aert & Wicherts, 2024) that corrects for selective reporting, which is one of the most common types of *p-*hacking (John, Loewenstein, & Prelec, 2012). Other more general approaches that are not restricted to correcting for only one type of *p*-hacking were also proposed. One approach is the Meta-Analysis Instrumental Variable Estimator (MAIVE; Irsova, Bom, Havránek, & Rachinger, 2024), which uses the inverse of the sample size as an instrumental variable for the standard error of a study in a meta-regression model to take into account that *p*-hacking likely affects the standard error. In a simulation study, MAIVE yielded less biased estimates than meta-analysis methods that do not correct for *p*-hacking in situations where primary studies included control variables that resulted in a statistically significant effect. Other approaches are the right-truncated meta-analysis (RTMA; Mathur, 2024b) and the meta-analysis of only the statistically nonsignificant primary studies (MAN; Mathur, 2024a; Mathur, 2024b). Both methods are based on the assumption that statistically nonsignificant primary studies are not *p-*hacked and therefore not biased. RTMA fits a right-truncated normal distribution to only the statistically nonsignificant primary studies to estimate the average effect size and between-study variance in true effect size of all studies (i.e., nonsignificant and significant studies). MAN is a meta-analysis based on only the statistically nonsignificant primary studies. As different types of *p*-hacking have different effects on effect size estimation (e.g., van Aert et al., 2016), future research may focus on studying which meta-analysis method performs best under different types of *p*-hacking.

To conclude, scientific progress can best be achieved by using meta-analysis (Cumming, 2008), but this progress is hampered by publication bias causing false positive and overestimated effect sizes. Hence, there is a need for methods that can accurately estimate the effect size and between-study variance in a meta-analysis in the presence of publication bias. We have introduced the new *p*-uniform* for this purpose that has been found to be a substantial improvement over *p*-uniform. Furthermore, *p-*uniform* appeared to be a viable alternative to 3PSM, because it is a more parsimonious model and showed comparable performance to 3PSM in simulation studies.

## Data Availability

Data of the applications are available via https://osf.io/9k3bp?view_only=c17ce4ec24b748e7b2dff33dcd42942e.

## Footnotes

1 Simonsohn et al. (2014) argue that *p*-curve accurately estimates effect size in the presence of heterogeneity, because it estimates the average true effect size of exactly the studies included in a meta-analysis. This is in contrast to the conventional random-effects meta-analysis model where the included studies are assumed to be a random sample of a population of studies and the estimated parameter is the mean of this population (Borenstein et al., 2009). See van Aert et al. (2016) for an elaborate discussion.

2 Effect sizes in *p-*uniform in its current implementation can be estimated using six different methods: maximum likelihood, and using the Irwin-Hall distribution, Fisher’s test, the Kolmogorov-Smirnov test, Anderson-Darling test, and a variant of Fisher’s test, $-\sum_{i=1}^{k}\mathrm{ln}(1-{q}_{i})$. All methods are implemented in the R package “puniform.”

3 We conducted an additional simulation study to examine whether varying the primary study’s sample size influenced the results (R code https://osf.io/d4tq9?view_only=c17ce4ec24b748e7b2dff33dcd42942e). The same variables were varied in this simulation study as in the one described above where was made sure that all simulated meta-analyses contained at least one statistically (non)significant effect size. The only difference between the two simulation studies was that *k* was fixed to 30 rather than systematically varied. Sample sizes were varied such that the median sample size per group of the primary studies included in a meta-analysis was equal to 50; ten studies had 25 observations per group, eight had 50 per group, six had 100 per group, four had 150 per group, and two had 300 per group. We only report the results of this simulation study in the Online Supplemental Materials (https://osf.io/gycf2?view_only=c17ce4ec24b748e7b2dff33dcd42942e), because the results of this simulation study were close to those with fixed sample size.

4 Confidence intervals of the random-effects model for *μ* were computed using the adjustment proposed by Hartung and Knapp (Hartung, 1999; Hartung & Knapp, 2001a, 2001b) and Sidik and Jonkman (Sidik & Jonkman, 2002), because coverage probability after applying this adjustment is closer to the nominal coverage rate (IntHout et al., 2014; Röver et al., 2015; Wiksten et al., 2016). Veroniki et al. (2016) reviewed existing methods to compute a confidence interval for *τ*^2^ and concluded that the *Q*-profile method (Viechtbauer, 2007b) and generalized *Q*-statistic method (Jackson, 2013) are the two methods with the best statistical properties. We decided to include the *Q*-profile method in our simulations, because this method does not require arbitrary choices with respect to the primary study’s weights as compared to the generalized *Q*-statistic method.

## Code availability

R codes of the simulation studies are available in the OSF repository of the paper: https://osf.io/ebq6m/?view_only=c17ce4ec24b748e7b2dff33dcd42942e.

## References

[CR1] Agresti, A. (2013). *Categorical data analysis*. Wiley-Interscience.

[CR2] Andrews, I., & Kasy, M. (2019). Identification of and correction for publication bias. *American Economic Review,**109*(8), 2766–2794. 10.1257/aer.20180310

[CR3] Appelbaum, M., Cooper, H., Kline, R. B., Mayo-Wilson, E., Nezu, A. M., & Rao, S. M. (2018). Journal article reporting standards for quantitative research in psychology: The APA Publications and Communications Board task force report. *The American psychologist,**73*(1), 3–25. 10.1037/amp0000191 29345484 10.1037/amp0000191

[CR4] Augusteijn, H. E. M., van Aert, R. C. M., & van Assen, M. A. L. M. (2019). The effect of publication bias on the Q test and assessment of heterogeneity. *Psychological Methods,**24*(1), 116–134. 10.1037/met0000197 30489099 10.1037/met0000197

[CR5] Bakker, M., van Dijk, A., & Wicherts, J. M. (2012). The rules of the game called psychological science. *Perspectives on Psychological Science,**7*(6), 543–554. 10.1177/1745691612459060 26168111 10.1177/1745691612459060

[CR6] Bangert-Drowns, R. L., Hurley, M. M., & Wilkinson, B. (2004). The effects of school-based writing-to-learn interventions on academic achievement: A meta-analysis. *Review of Educational Research,**74*(1), 29–58. 10.3102/00346543074001029

[CR7] Bartoš, F., Maier, M., Wagenmakers, E.-J., Doucouliagos, H., & Stanley, T. D. (2023). Robust bayesian meta-analysis: Model-averaging across complementary publication bias adjustment methods. *Research Synthesis Methods,**14*(1), 99–116. 10.1002/jrsm.1594 35869696 10.1002/jrsm.1594 PMC10087723

[CR8] Bartoš, F., Maier, M., Wagenmakers, E.-J., Nippold, F., Doucouliagos, H., Ioannidis, J. P. A., Wagenmakers, Eric‐Jan., Otte, Willem M.., Sladekova, Martina, Deresssa, Teshome K.., Bruns, Stephan B.., Fanelli, Daniele, & Stanley, T. D. (2024). Footprint of publication selection bias on meta-analyses in medicine, environmental sciences, psychology, and economics. *Research Synthesis Methods,**15*(3), 500–511. 10.1002/jrsm.1703 38327122 10.1002/jrsm.1703

[CR9] Borenstein, M., Hedges, L. V., Higgins, J. P. T., & Rothstein, H. R. (2009). *Introduction to meta-analysis*. Chichester, UK: John Wiley & Sons, Ltd.

[CR10] Carter, E. C., Schönbrodt, F. D., Gervais, W. M., & Hilgard, J. (2019). Correcting for bias in psychology: A comparison of meta-analytic methods. *Advances in Methods and Practices in Psychological Science,**2*(2), 115–144. 10.1177/2515245919847196

[CR11] Cleary, R. J., & Casella, G. (1997). An application of Gibbs sampling to estimation in meta-analysis: Accounting for publication bias. *Journal of Educational and Behavioral Statistics,**22*(2), 141–154.

[CR12] Coburn, K. M., & Vevea, J. L. (2015). Publication bias as a function of study characteristics. *Psychological Methods,**20*(3), 310–330. 10.1037/met0000046 26348731 10.1037/met0000046

[CR13] Coburn, K. M., & Vevea, J. L. (2016). weightr: Estimating weight-function models for publication bias. Retrieved from https://CRAN.R-project.org/package=weightr

[CR14] Cohen, J. (1988). *Statistical power analysis for the behavioral sciences* (2nd ed.). Lawrence Erlbaum Associates.

[CR15] Cohen, J. (1990). Things i have learned (so far). *American Psychologist,**45*(12), 1304–1312.

[CR16] Cooper, H., DeNeve, K., & Charlton, K. (1997). Finding the missing science: The fate of studies submitted for review by a human subjects committee. *Psychological Methods,**2*(4), 447–452. 10.1037/1082-989X.2.4.447

[CR17] Copas, J. B., & Shi, J. Q. (2000). Meta-analysis, funnel plots and sensitivity analysis. *Biostatistics,**1*(3), 247–262. 10.1093/biostatistics/1.3.247 12933507 10.1093/biostatistics/1.3.247

[CR18] Coursol, A., & Wagner, E. E. (1986). Effect of positive findings on submission and acceptance rates: A note on meta-analysis bias. *Professional Psychology: Research and Practice,**17*(2), 136–137. 10.1037/0735-7028.17.2.136

[CR19] Cumming, G. (2008). Replication and p intervals: P values predict the future only vaguely, but confidence intervals do much better. *Perspectives on Psychological Science,**3*(4), 286–300. 10.1111/j.1745-6924.2008.00079.x 26158948 10.1111/j.1745-6924.2008.00079.x

[CR20] Dickersin, K., & Rennie, D. (2003). Registering clinical trials. *JAMA,**290*(4), 516–523. 10.1001/jama.290.4.516 12876095 10.1001/jama.290.4.516

[CR21] Duval, S., & Tweedie, R. L. (2000). A nonparametric “trim and fill” method of accounting for publication bias in meta-analysis. *Journal of the American Statistical Association,**95*(449), 89–98. 10.1080/01621459.2000.10473905

[CR22] Duval, S., & Tweedie, R. L. (2000). Trim and fill: A simple funnel-plot-based method of testing and adjusting for publication bias in meta-analysis. *Biometrics,**56*(2), 455–463. 10877304 10.1111/j.0006-341x.2000.00455.x

[CR23] Eddelbuettel, D. (2013). *Seamless R and C++ integration with Rcpp*. Springer.

[CR24] Fanelli, D. (2010). “Positive” results increase down the hierarchy of the sciences. *PLoS One,**5*(4), Article e10068. 10.1371/journal.pone.0010068 20383332 10.1371/journal.pone.0010068 PMC2850928

[CR25] Fanelli, D. (2012). Negative results are disappearing from most disciplines and countries. *Scientometrics,**90*(3), 891–904. 10.1007/s11192-011-0494-7

[CR26] Field, A. P., & Gillett, R. (2010). How to do a meta-analysis. *British Journal of Mathematical and Statistical Psychology,**63*(3), 665–694. 10.1348/000711010X502733 20497626 10.1348/000711010X502733

[CR27] Fisher, R. A. (1925). *Statistical methods for research workers* (1st ed.). Oliver & Boyd.

[CR28] Franco, A., Malhotra, N., & Simonovits, G. (2014). Publication bias in the social sciences: Unlocking the file drawer. *Science,**345*(6203), 1502–1505. 10.1126/science.1255484 25170047 10.1126/science.1255484

[CR29] Hartung, J. (1999). An alternative method for meta-analysis. *Biometrical Journal,**41*(8), 901–916.

[CR30] Hartung, J., & Knapp, G. (2001). On tests of the overall treatment effect in meta-analysis with normally distributed responses. *Statistics in Medicine,**20*(12), 1771–1782. 10.1002/sim.791 11406840 10.1002/sim.791

[CR31] Hartung, J., & Knapp, G. (2001). A refined method for the meta-analysis of controlled clinical trials with binary outcome. *Statistics in Medicine,**20*(24), 3875–3889. 10.1002/sim.1009 11782040 10.1002/sim.1009

[CR32] Hedges, L. V. (1981). Distribution theory for Glass’s estimator of effect size and related estimators. *Journal of Educational Statistics,**6*(2), 107–128.

[CR33] Hedges, L. V. (1983). A random effects model for effect sizes. *Psychological Bulletin,**93*(2), 388–395. 10.1037/0033-2909.93.2.388

[CR34] Hedges, L. V. (1984). Estimation of effect size under nonrandom sampling: The effects of censoring studies yielding statistically insignificant mean differences. *Journal of Educational Statistics,**9*(1), 61–85.

[CR35] Hedges, L. V. (1992). Modeling publication selection effects in meta-analysis. *Statistical Science, 7*(2), 246–255.

[CR36] Hedges, L. V., & Vevea, J. L. (1996). Estimating effect size under publication bias: Small sample properties and robustness of a random effects selection model. *Journal of Educational and Behavioral Statistics,**21*(4), 299–332.

[CR37] Hedges, L. V., & Vevea, J. L. (2005). Selection method approaches. In H. R. Rothstein, A. J. Sutton, & M. Borenstein (Eds.), *Publication bias in meta-analysis: Prevention, assessment, and adjustments*. Chichester: UK: Wiley.

[CR38] Higgins, J. P. T., Thompson, S. G., Deeks, J. J., & Altman, D. G. (2003). Measuring inconsistency in meta-analyses. *British Medical Journal,**327*(7414), 557–560. 10.1136/bmj.327.7414.557 12958120 10.1136/bmj.327.7414.557 PMC192859

[CR39] Hong, S., & Reed, W. R. (2021). Using Monte Carlo experiments to select meta-analytic estimators. *Research Synthesis Methods,**12*(2), 192–215. 10.1002/jrsm.1467 33150663 10.1002/jrsm.1467 PMC8074967

[CR40] IntHout, J., Ioannidis, J. P., & Borm, G. F. (2014). The Hartung-Knapp-Sidik-Jonkman method for random effects meta-analysis is straightforward and considerably outperforms the standard DerSimonian-Laird method. *BMC Medical Research Methodology*. 10.1186/1471-2288-14-25 24548571 10.1186/1471-2288-14-25 PMC4015721

[CR41] Irsova, Z., Bom, P. R. D., Havranek, T., & Rachinger, H. (2025). Spurious precision in meta-analysis of observational research. *Nature Communications*, *16*(1), 8454. 10.1038/s41467-025-63261-0 10.1038/s41467-025-63261-0 PMC12475282 41006270

[CR42] Iyengar, S., & Greenhouse, J. B. (1988a). Selection models and the file drawer problem. *Statistical Science, 3*(1), 109–117. 10.1214/ss/1177013012

[CR43] Iyengar, S., & Greenhouse, J. B. (1988b). Selection models and the file drawer problem: Rejoinder. *Statistical Science, 3*(1), 133 135.

[CR44] Jackson, D. (2006). The implications of publication bias for meta-analysis’ other parameter. *Statistics in Medicine,**25*(17), 2911–2921. 10.1002/sim.2293 16345059 10.1002/sim.2293

[CR45] Jackson, D. (2007). Assessing the implications of publication bias for two popular estimates of between-study variance in meta-analysis. *Biometrics,**63*(1), 187–193. 10.1111/j.1541-0420.2006.00663.x 17447944 10.1111/j.1541-0420.2006.00663.x

[CR46] Jackson, D. (2013). Confidence intervals for the between-study variance in random effects meta-analysis using generalised Cochran heterogeneity statistics. *Research Synthesis Methods,**4*(3), 220–229. 10.1002/jrsm.1081 26053842 10.1002/jrsm.1081

[CR47] John, L. K., Loewenstein, G., & Prelec, D. (2012). Measuring the prevalence of questionable research practices with incentives for truth telling. *Psychological Science,**23*(5), 524–532. 10.1177/0956797611430953 22508865 10.1177/0956797611430953

[CR48] Kepes, S., Banks, G. C., McDaniel, M., & Whetzel, D. L. (2012). Publication bias in the organizational sciences. *Organizational Research Methods,**15*(4), 624–662. 10.1177/1094428112452760

[CR49] Kicinski, M. (2013). Publication bias in recent meta-analyses. *PLoS One*. 10.1371/journal.pone.0081823 24363797 10.1371/journal.pone.0081823 PMC3868709

[CR50] Kraemer, H. C., Gardner, C., Brooks, J., & Yesavage, J. A. (1998). Advantages of excluding underpowered studies in meta-analysis: Inclusionist versus exclusionist viewpoints. *Psychological Methods,**3*(1), 23–31. 10.1037/1082-989X.3.1.23

[CR51] Lane, D. M., & Dunlap, W. P. (1978). Estimating effect size: Bias resulting from the significance criterion in editorial decisions. *British Journal of Mathematical and Statistical Psychology,**31*, 107–112.

[CR52] Langan, D., Higgins, J. P. T., & Simmonds, M. (2016). Comparative performance of heterogeneity variance estimators in meta-analysis: A review of simulation studies. *Research Synthesis Methods,**8*(2), 181–198. 10.1002/jrsm.1198 27060925 10.1002/jrsm.1198

[CR53] Mathur, M. B. (2024). Assessing robustness to worst case publication bias using a simple subset meta-analysis. *BMJ (Clinical research ed.),**384*, Article e076851. 10.1136/bmj-2023-076851 38490665 10.1136/bmj-2023-076851 PMC10941077

[CR54] Mathur, M. B. (2024). P-hacking in meta-analyses: A formalization and new meta-analytic methods. *Research Synthesis Methods,**15*(3), 483–499. 10.1002/jrsm.1701 38273211 10.1002/jrsm.1701 PMC11042997

[CR55] McShane, B. B., Böckenholt, U., & Hansen, K. T. (2016). Adjusting for publication bias in meta-analysis: An evaluation of selection methods and some cautionary notes. *Perspectives on Psychological Science,**11*(5), 730–749. 10.1177/1745691616662243 27694467 10.1177/1745691616662243

[CR56] Niemeyer, H., van Aert, R. C. M., Schmid, S., Uelsmann, D., Knaevelsrud, C., & Schulte-Herbrueggen, O. (2020). Publication bias in meta-analyses of posttraumatic stress disorder interventions. *Meta-Psychology, 4*. 10.15626/MP.2018.884

[CR57] Paule, R. C., & Mandel, J. (1982). Consensus values and weighting factors. *Journal of Research of the National Bureau of Standards,**87*(5), 377–385. 34566088 10.6028/jres.087.022 PMC6768160

[CR58] Pawitan, Y. (2013). *In all likelihood: Statistical modelling and inference using likelihood*. OUP Oxford.

[CR59] R Core Team. (2024). R: A language and environment for statistical computing. Retrieved from http://www.R-project.org/

[CR60] Rabelo, A. L. A., Keller, V. N., Pilati, R., & Wicherts, J. M. (2015). No effect of weight on judgments of importance in the moral domain and evidence of publication bias from a meta-analysis. *PLoS One,**10*(8), Article e0134808. 10.1371/journal.pone.0134808 26241042 10.1371/journal.pone.0134808 PMC4524628

[CR61] Rothstein, H. R., Sutton, A. J., & Borenstein, M. (2005). Publication bias in meta-analysis. In H. R. Rothstein, A. J. Sutton, & M. Borenstein (Eds.), *Publication bias in meta-analysis: Prevention, assessment and adjustments*. Chichester, UK: Wiley.

[CR62] Röver, C., Knapp, G., & Friede, T. (2015). Hartung-knapp-sidik-jonkman approach and its modification for random-effects meta-analysis with few studies. *BMC Medical Research Methodology*. 10.1186/s12874-015-0091-1 26573817 10.1186/s12874-015-0091-1 PMC4647507

[CR63] Sidik, K., & Jonkman, J. N. (2002). A simple confidence interval for meta-analysis. *Statistics in Medicine,**21*(21), 3153–3159. 10.1002/sim.1262 12375296 10.1002/sim.1262

[CR64] Simes, R. J. (1986). Publication bias: The case for an international registry of clinical trials. *Journal of Clinical Oncology,**4*(10), 1529–1541. 3760920 10.1200/JCO.1986.4.10.1529

[CR65] Simmons, J. P., Nelson, L. D., & Simonsohn, U. (2011). False-positive psychology: Undisclosed flexibility in data collection and analysis allows presenting anything as significant. *Psychological Science,**22*(11), 1359–1366. 10.1177/0956797611417632 22006061 10.1177/0956797611417632

[CR66] Simonsohn, U., Nelson, L. D., & Simmons, J. P. (2014). P-curve and effect size: Correcting for publication bias using only significant results. *Perspectives on Psychological Science,**9*(6), 666–681. 10.1177/1745691614553988 26186117 10.1177/1745691614553988

[CR67] Simonsohn, U., Simmons, J. P., & Nelson, L. D. (2017, December 20). Why p-curve excludes ps>.05 [Web log message]. Retrieved from http://datacolada.org/61

[CR68] van Aert, R. C. M. (2022). puniform: Meta-analysis methods correcting for publication bias. (Version 0.2.5). Retrieved from https://CRAN.R-project.org/package=puniform

[CR69] van Aert, R. C. M., & Wicherts, J. M. (2024). Correcting for outcome reporting bias in a meta-analysis: A meta-regression approach. *Behavior Research Methods,**56*(3), 1994–2012. 10.3758/s13428-023-02132-2 37540470 10.3758/s13428-023-02132-2 PMC10991008

[CR70] van Aert, R. C. M., Wicherts, J. M., & van Assen, M. A. L. M. (2016). Conducting meta-analyses on p-values: Reservations and recommendations for applying p-uniform and p-curve. *Perspectives on Psychological Science,**11*(5), 713–729. 10.1177/1745691616650874 27694466 10.1177/1745691616650874 PMC5117126

[CR71] van Aert, R. C. M., Wicherts, J. M., & van Assen, M. A. L. M. (2019). Publication bias examined in meta-analyses from psychology and medicine: A meta-meta-analysis. *PLoS One*. 10.1371/journal.pone.0215052 30978228 10.1371/journal.pone.0215052 PMC6461282

[CR72] van Assen, M. A. L. M., van Aert, R. C. M., & Wicherts, J. M. (2015). Meta-analysis using effect size distributions of only statistically significant studies. *Psychological Methods,**20*(3), 293–309. 10.1037/met0000025 25401773 10.1037/met0000025

[CR73] van Erp, S. J., Verhagen, J., Grasman, R. P. P. P., & Wagenmakers, E.-J. (2017). Estimates of between-study heterogeneity for 705 meta-analyses reported in Psychological Bulletin from 1990-2013. *Journal of Open Psychology Data, 5*(1). 10.5334/jopd.33

[CR74] Veroniki, A. A., Jackson, D., Viechtbauer, W., Bender, R., Bowden, J., Knapp, G., & Salanti, G. (2016). Methods to estimate the between-study variance and its uncertainty in meta-analysis. *Research Synthesis Methods,**7*(1), 55–79. 10.1002/jrsm.1164 26332144 10.1002/jrsm.1164 PMC4950030

[CR75] Vevea, J. L., & Woods, C. M. (2005). Publication bias in research synthesis: Sensitivity analysis using a priori weight functions. *Psychological Methods,**10*(4), 428–443. 10.1037/1082-989X.10.4.428 16392998 10.1037/1082-989X.10.4.428

[CR76] Viechtbauer, W. (2005). Bias and efficiency of meta-analytic variance estimators in the random-effects model. *Journal of Educational and Behavioral Statistics,**30*(3), 261–293. 10.3102/10769986030003261

[CR77] Viechtbauer, W. (2007). Approximate confidence intervals for standardized effect sizes in the two-independent and two-dependent samples design. *Journal of Educational and Behavioral Statistics,**32*(1), 39–60. 10.3102/1076998606298034

[CR78] Viechtbauer, W. (2007). Confidence intervals for the amount of heterogeneity in meta-analysis. *Statistics in Medicine,**26*(1), 37–52. 10.1002/sim.2514 16463355 10.1002/sim.2514

[CR79] Viechtbauer, W. (2010). Conducting meta-analyses in R with the metafor package. *Journal of Statistical Software,**36*(3), 1–48. 10.18637/jss.v036.i03

[CR80] Wicherts, J. M., Veldkamp, C. L. S., Augusteijn, H. E. M., Bakker, M., van Aert, R. C. M., & van Assen, M. A. L. M. (2016). Degrees of freedom in planning, running, analyzing, and reporting psychological studies: A checklist to avoid p-hacking. *Frontiers in Psychology, 7*(1832). 10.3389/fpsyg.2016.01832 10.3389/fpsyg.2016.01832 PMC5122713 27933012

[CR81] Wiksten, A., Rücker, G., & Schwarzer, G. (2016). Hartung-knapp method is not always conservative compared with fixed-effect meta-analysis. *Statistics in Medicine,**35*(15), 2503–2515. 10.1002/sim.6879 26842654 10.1002/sim.6879

